# YBX1-interacting small RNAs and *RUNX2* can be blocked in primary bone cancer using CADD522

**DOI:** 10.1016/j.jbo.2023.100474

**Published:** 2023-03-05

**Authors:** Darrell Green, Archana Singh, Victoria L. Tippett, Luke Tattersall, Karan M. Shah, Chileleko Siachisumo, Nicole J. Ward, Paul Thomas, Simon Carter, Lee Jeys, Vaiyapuri Sumathi, Iain McNamara, David J. Elliott, Alison Gartland, Tamas Dalmay, William D. Fraser

**Affiliations:** a Biomedical Research Centre, Norwich Medical School, University of East Anglia, Norwich, UK; b School of Biological Sciences, University of East Anglia, Norwich, UK; c The Mellanby Centre for Musculoskeletal Research, Department of Oncology and Metabolism, The University of Sheffield, UK; d Biosciences Institute, Newcastle University, Newcastle, UK; e Henry Wellcome Laboratory for Cell Imaging, Faculty of Science, University of East Anglia, Norwich, UK; f Orthopaedic Oncology, Royal Orthopaedic Hospital, Birmingham, UK; g Musculoskeletal Pathology, University Hospitals Birmingham, Royal Orthopaedic Hospital, Birmingham, UK; h Orthopaedics & Trauma, Norfolk and Norwich University Hospital, Norwich, UK; i Clinical Biochemistry, Diabetes and Endocrinology, Norfolk and Norwich University Hospital, Norwich, UK

**Keywords:** miRNA, tRF, small RNA, bone cancer, CADD522, CADD522, computer aided drug design molecule 522, CI, confidence interval, CNV, copy number variant, CS, chondrosarcoma, CTC, circulating tumour cell, DE, differentially expressed, ES, Ewing sarcoma, HD, high definition, HR, hazard ratio, iCLIP, individual nucleotide resolution cross-linking and immunoprecipitation, miRNA, microRNA, mRNA, messenger RNA, OS, osteosarcoma, piRNA, piwi interacting RNA, RBP, RNA binding protein, Rnl, T4 RNA ligase, RNU6-1, U6 small nuclear 1, ROI, region-of-interest, SNV, single nucleotide variant, sRNA, small RNA, SV, structural variant, tRNA, transfer RNA, tRF, transfer RNA fragment, ysRNA, Y RNA-derived sRNA

## Abstract

Primary bone cancer (PBC) comprises several subtypes each underpinned by distinctive genetic drivers. This driver diversity produces novel morphological features and clinical behaviour that serendipitously makes PBC an excellent metastasis model. Here, we report that some transfer RNA-derived small RNAs termed tRNA fragments (tRFs) perform as a constitutive tumour suppressor mechanism by blunting a potential pro-metastatic protein-RNA interaction. This mechanism is reduced in PBC progression with a gradual loss of tRNAGly^TCC^ cleavage into 5′ end tRF-Gly^TCC^ when comparing low-grade, intermediate-grade and high-grade patient tumours. We detected recurrent activation of miR-140 leading to upregulated *RUNX2* expression in high-grade patient tumours. Both tRF-Gly^TCC^ and *RUNX2* share a sequence motif in their 3′ ends that matches the YBX1 recognition site known to stabilise pro-metastatic mRNAs. Investigating some aspects of this interaction network, gain- and loss-of-function experiments using small RNA mimics and antisense LNAs, respectively, showed that ectopic tRF-Gly^TCC^ reduced *RUNX2* expression and dispersed 3D micromass architecture *in vitro*. iCLIP sequencing revealed YBX1 physical binding to the 3′ UTR of *RUNX2*. The interaction between YBX1, tRF-Gly^TCC^ and *RUNX2* led to the development of the RUNX2 inhibitor CADD522 as a PBC treatment. CADD522 assessment *in vitro* revealed significant effects on PBC cell behaviour. In xenograft mouse models, CADD522 as a single agent without surgery significantly reduced tumour volume, increased overall and metastasis-free survival and reduced cancer-induced bone disease. Our results provide insight into PBC molecular abnormalities that have led to the identification of new targets and a new therapeutic.

## 1 Introduction

Sarcomas are heterogeneous and clinically challenging bone and soft tissue cancers [1]. Sarcomas disproportionately affect children 10-fold when compared to adults. According to Public Health England’s children, teenager and young adult UK cancer statistics report 2021, sarcoma is the third commonest childhood cancer. Almost half of all sarcomas are primary bone cancer (PBC), which affects ∼ 6 per 10^6^ individuals annually worldwide [2], [3]. PBC typically arises either side of the knee or in the pelvis [4]. PBC is heterogeneous with subtypes underpinned by discrete genetic drivers leading to diverse morphological features and clinical behaviour. The two commonest PBC subtypes in children are osteosarcoma (OS) and Ewing sarcoma (ES). The commonest subtype in adults is chondrosarcoma (CS).

Paediatric and young adult OS is distinct from adult OS (>65 y). Adult OS mechanisms are consistent with other more common cancers involving life-long mutation accumulation (i.e. environment), as a secondary to chronic disease (e.g. Paget’s disease of bone) and unavoidable DNA replication errors (i.e. chance/luck) [5]. Paediatric OS similarly to other childhood cancers such as acute lymphoblastic leukaemia [6] has been associated with embryonic development errors. This association has led to the hypothesis that OS develops in two discrete steps. First, near ubiquitous *in utero* initiation by abnormal paternal imprinting at the 14q32 locus in a multipotent mesenchymal progenitor, which generates a pre-OS clone [7], [8]. Second, the postnatal acquisition of *TP53* or *RB1* structural variants by chromoplexy or chromothripsis in a partially differentiated osteoblast [9], [10]. This 14q32-*TP53*/*RB1* mutant does not enter apoptosis and is sufficient for tumorigenesis. Subsequent mutations such as *CDKN2A*, *MYC, BRCA* and *IGF* dictate metastasis and treatment response [11]. The 14q32 locus is also affected in some ES cases [12] but the more robustly reported ES driver is an in-frame *EWSR1* fusion with variable *ETS* transcription factors (∼85% *FLI1*, ∼10% *ERG*) [13]. *EWSR1-ETS* encode aberrant transcription factors with neomorphic features that massively reprogramme the transcriptome, epigenome and spliceosome; global changes that are partially mediated through EWSR1-ETS binding to GGAA microsatellites located at condensed chromatin regions that are converted to potent neo-enhancers [13]. Both OS and ES are high-grade at diagnosis with one in four cases presenting with detectable lung or bone metastases [2]. Standard of care involves non-specific combination chemotherapy and surgery. Five-year survival is poor at ∼ 50% [2], [4]. Neither OS nor ES have observed treatment or survival improvement for 45 years. These malignancies require fundamental molecular investigation to identify targets to disrupt disease progression, but their inherently aggressive phenotype has hampered tangible progress when compared to some other tumours. Novel approaches - and sometimes distant hypotheses - are required.

CS tumours in adults also comprise a 14q32 lesion [14] but the transformative driver remains unknown, though some CS tumours harbour mutations in *IDH1* or *IDH2* [15], which supports CS diagnosis. Unlike OS and ES that are high-grade at diagnosis, CS presents discrete morphological features and clinical behaviour at low, intermediate and high-grade. Although PBC subtypes are distinct diseases comprising their own biology and clinical phenotype, CS as the more indolent PBC subtype provides a proof-of-concept model for disease progression. Not all but some datasets generated in CS may provide insight into fundamental events and/or early metastatic hallmarks underpinning high-grade OS and ES, which otherwise would not be detected due to their high-grade diagnosis. Some of these hallmarks will be transcriptome-based components where differentially expressed (DE) non-coding RNAs are detected by RNA-seq [11] but are not detected using whole genome sequencing.

Non-coding RNAs are powerful regulators of gene expression and perform several roles in cancer progression [16]. Small RNAs (sRNAs) 19–32 nucleotides (nt) in length are a diverse set of non-coding RNAs that regulate gene expression through transcriptional and post-transcriptional gene silencing [17]. Human sRNA classes include microRNAs (miRNAs), piwi-interacting RNAs (piRNAs) and Y RNA-derived sRNAs (ysRNAs) [18], [19]. Transfer RNAs (tRNAs) are viewed as inert contributors to gene expression but have recently been implicated in post-transcriptional gene silencing [20] (reviewed in [21]). Full length tRNAs (76–90 nt) causally impact disease progression by selective ribosome occupancy [22]. Mature tRNA endoribonuclease cleavage produces tRNA-derived sRNAs termed tRNA fragments (tRFs) (21–32 nt) [23]. TRFs that contain a (C/G)CU(C/G/U)(C/U)C sequence motif at the 3′ end are known to interact with the RNA binding protein (RBP) YBX1 to act as a decoy to prevent YBX1 from stabilising pro-metastatic messenger RNAs (mRNAs) for translation in breast cancer [24].

sRNA identification by library construction and next generation sequencing or sRNA-seq has historically been biased for sRNA sequences that readily anneal to 5′ and 3′ adapters during the protocol ligation steps [25], [26], [27]. Bias exists because the adapters used in commercially available kits are fixed 26 nt RNA (5′) and 23 nt DNA (3′) sequences. Fixed adapter sequences are disadvantaged because sRNAs with a low annealing efficiency to the adapters are less likely to be ligated and less probable to be sequenced. Most sRNA library protocols implement adenylated 3′ adapter ligation using a truncated T4 RNA ligase 2 (Rnl2) to ligate the sRNA 3́' hydroxyl group to the DNA adapter 5́' phosphate group. The single stranded RNA-DNA hybrid is then ligated to a 5′ RNA adapter using T4 RNA ligase 1 (Rnl1). The double ligated product is then converted to double stranded cDNA and amplified by PCR [27]. We previously tested cDNA libraries to survey Rnl1/2 sequence preference using libraries comprising randomised nucleotides such that all possible sequences were presumed to have similar concentrations [28]. This study revealed important Rnl1/2 ligation bias that led us to develop high definition (HD) adapters [28]. HD adapters contain four randomly assigned nucleotides at the ligating ends of TruSeq adapters providing a pool of 2^8^ sequences that significantly increases the annealing efficiency between sRNAs and adapters [28], [29]. HD adapters reduced sRNA-seq bias with increased low abundance sRNA representation and allowed for new sRNAs to be identified [29].

Here, we used HD adapters to characterise the sRNA landscape in CS patient samples (low to high-grade) as a proof-of-concept model for PBC progression. We addressed the hypothesis that some of the progression mechanisms and/or DE targets will overlap between the PBC subtypes and therefore datasets will be applicable to high-grade OS and ES when developing new therapies.

## 2 Materials and methods

### 2.1 Patient samples

The UEA Faculty of Medicine and Health Sciences Research Ethics Committee approved human sample collection and study (2013/2014 – 22 HT). A consultant histopathologist confirmed CS grade at biopsy and resection using World Health Organisation guidelines. Our patient series included control cartilage tissue from the femoral neck (n = 6), low-grade CS (n = 6), intermediate-grade CS (n = 3) and high-grade CS (n = 4). N numbers were all of the available samples that came through our clinic in a one-year period. All individuals provided written informed consent to donate tissue to this study. Sample data are summarised in Suppl. File 1.

### 2.2 Cell lines

SW1353 (RRID: CVCL_0543; human CS), 143B (RRID: CVCL_2270; human OS) and TC71 (RRID: CVCL_S882; human ES) cells were obtained from the American Type Culture Collection and Public Health England. For *in vivo* experiments, 143B and TC71 cells were modified by transfecting with the GFP-luciferase tagged LVP02 lentivirus (AMSBIO). Adding the luciferase tag had no observable or measurable effect on cell phenotype, which was assessed during this study and previously [30]. Cells were authenticated by STR profiling (Suppl. File 2). All experiments were performed with *Mycoplasma*-free cells. Cells were cultured in DMEM (Thermo Fisher Scientific) or IMDM (Thermo Fisher Scientific) containing 10% (v/v) FBS and 1% (v/v) penicillin/streptomycin at 37 °C and 5% CO_2_.

### 2.3 Proliferation assays

1 × 10^3^ cells in 100 µL medium were seeded per well into 96-well plates. PBS was added to the outer wells to prevent evaporation. All plates were then incubated for 24 h. CADD522 from a 10 mM stock in DMSO was added in 100 nM, 1 µM, 10 µM and 100 µM concentrations and incubated for 72 h before measuring cell viability and proliferation using the WST-1 assay (Abcam). Data was presented as relative to the control, which was set to 100% viability. Experiments were performed in quadruplicates in three independent experiments.

### 2.4 sRNA-seq

CS samples were homogenised under liquid nitrogen. Total RNA was extracted using the miRCURY RNA isolation kit (Exiqon) according to the manufacturer’s instructions. RNA concentration and integrity was measured on the NanoDrop 8000 (Thermo Fisher Scientific) as well as visual assessment by agarose gel electrophoresis. RNA was stored at −80 °C. sRNA libraries were constructed using 1 µg RNA ligated to 3′ and 5′ HD adapters [29]. Ligated RNA products were reverse transcribed to cDNA and amplified by PCR. cDNA products expected to contain 19–32 bp inserts were purified by 8% PAGE and ethanol precipitation. Finally, 50 bp single end sequencing was performed on a HiSeq 2500 (Illumina).

### 2.5 Bioinformatics

FASTQ files were converted to FASTA. Reads containing unassigned nucleotides were excluded. HD adapter signatures (four assigned nt at the ligating ends) plus the 3′ adapter were trimmed using perfect sequence matching to the first 8 nt of the 3′ HiSeq 2500 adapter (TGGAATTC). sRNAs were mapped full length with 0 mismatches to the human genome (v38) and corresponding annotations using PatMaN [31]. The latest set of human miRNAs and tRFs were downloaded from miRBase (v22) and tRFdb (v1) [32], [33]. Normalisation and DE analysis was performed using DESeq2 (v1.2.10) [34]. Independent filtering was used to remove low abundance transcripts in normalised counts. sRNAs were considered DE if they had a p value < 0.05, false discovery rate (FDR) < 5% according to the Benjamini–Hochberg procedure [35] and a log_2_ offset fold change > 1 [11]. sRNA-seq coverage and quality statistics for each sample are summarised in Suppl. File 1.

### 2.6 Northern blot

We loaded 1 µg RNA mixed with Ambion gel loading buffer II (Thermo Fisher Scientific) and performed 16% urea PAGE. The gel was run at 120 V for 2 h in 0.5X TBE. RNA was transferred to a Hybond-NX (GE Healthcare) membrane using semidry transfer conditions at 250 mA for 45 min. RNA was then cross-linked to the membrane by adding 5 mL cross-linking solution adjusted to pH 8 (12 mL water, 122.5 mL 12.5 M 1-methylimidazole, 10 mL 1 M hydrochloric acid and 0.373 g 1-ethyl-3-(3-dimethylaminopropyl) carbodiimide) and incubating at 60 °C for 1 h in saran wrap. For each sRNA the membrane was pre-hybridised with ultra-hyb-oligo buffer (Thermo Fisher Scientific) at 37 °C for 1 h. We then incubated a mixture of 10 µL water, 4 µL 5X polynucleotide kinase forward buffer (New England Biolabs), 2 µL 10 mM DNA antisense oligonucleotide, 1 µL T4 polynucleotide kinase (New England Biolabs) and 3 µL γ-ATP at 37 °C for 1 h. The membrane was incubated in this buffer rotating at 37 °C overnight. Membrane was then washed three times in 0.2X SSC, 0.1% SDS before exposing on a phosphorimaging screen in a radioactive cassette (Fujifilm) followed by imaging on the FX Pro Plus molecular imager (Bio-Rad). The membrane was re-probed using antisense DNA oligos (Sigma Aldrich). RNA, U6 small nuclear 1 (*RNU6-1*) was used as the loading control.

### 2.7 Micromass cultures

Micromass cultures were constructed as previously described [36]. Briefly, 80% confluent monolayer cell cultures were released by trypsin-EDTA and resuspended in medium at a density of 4 × 10^4^ cells/mL. Micromass’ were achieved by pipetting 20 µL cell suspension into individual wells of 24 well plates. Following a 3 h intermolecular cohesion period, 500 µL medium was gently added. Cultures formed 3D spheres over 24 h.

### 2.8 sRNA/LNA transfection

Once 3D micromass cultures had formed, tRF mimics (RNA), tRF inhibitors (LNA), miRNA mimics (RNA) and miRNA inhibitors (LNA) were transfected using Lipofectamine 3000 (Thermo Fisher Scientific) with 50 nM RNA or LNA (Exiqon). Cell entry confirmation at 50 nM concentration was determined after performing transfection assessment with FAM-labelled sequences. After 6 h incubation, antibiotic-free opti-MEM medium containing mimics or inhibitors was replaced with fresh DMEM medium. Micromass’ were subjected to histology, microscopy and qPCR after 48 h. Transfections were performed in triplicate in five independent experiments.

### 2.9 Microscopy

Micromass’ were fixed in 3.7% formaldehyde and 0.2% Triton-X 100. DAPI and F-actin staining was visualised using an Axiovert 200 M (Zeiss) with an Axiocam MRm CCD (Zeiss) under AxioVision control. DAPI was excited at 365 nm and emission was collected between 420 and 470 nm using Zeiss filter set #49. Texas Red X Phalloidin (F-actin) was excited at 565 nm and emission was collected through a 615 nm long-pas (LP) filter using Zeiss filter set #00. Nuclear DAPI staining was segmented with the number/size of the nuclei quantified using ImageJ [37].

### 2.10 qPCR

Transcriptional profiling was performed using qPCR [38]. We also isolated total RNA from 3D cultures transfected with a tRF-Gly^TCC^ mimic (n = 6). Taqman gene expression assays (Thermo Fisher Scientific) were used following the manufacturer’s protocol. *ACTB* was used as a reference to normalise gene expression. qPCR was performed in a final volume of 10 µL using a 7500 Fast Real Time PCR instrument (Applied Biosystems) under the following cycling conditions: 95 °C for 20 s, 40 cycles at 95 °C for 3 s and 60 °C for 30 s in three independent experiments. Data is shown as mean ± SD.

### 2.11 sRNA mimic and LNA antisense sequences

Negative control mimic, GAUGGCAUUCGUCAGUUCUA; negative control inhibitor, TAACACGTCTATACGCCCA; tRF-Gly^TCC^ mimic, GCGUUGGUGGUAUAGUGGUGAGCAUAGCUGCC; tRF-Lys^TTT^ mimic, GCCCGGAUAGCUCAGUCGGUAGAGCAUCAGACU; tRF-Asn^GTT^ mimic, UUGGUGGUUCGAGCCCACCCAGGGACG; tRF-Gly^TCC^ inhibitor, TATGCTCACCACTATACCAC; tRF-Lys^TTT^ inhibitor, CTCTACCGACTGAGCTATCC; tRF-Asn^GTT^ inhibitor, GTGGGCTCGAACCACCAA; miR-140-3p mimic, TACCACAGGGTAGAACCACGG; miR-320a mimic, AAAAGCTGGGTTGAGAGGGCGA; miR-486-5p mimic, TCCTGTACTGAGCTGCCCCGAG; miR-140-3p inhibitor, CGTGGTTCTACCCTGTGGT; miR-320a inhibitor, CGCCCTCTCAACCCAGCTTT; miR-486-5p inhibitor, TCGGGGCAGCTCAGTACAG.

### 2.12 Individual nucleotide resolution cross-linking and immunoprecipitation (iCLIP)

SW1353 cells were irradiated with 150 mJ/cm^2^ UV at 254 nm. Cell pellets were resuspended in lysis buffer and sonicated in the Bioruptor (Diagenode) for 10 cycles with alternating 30 s on/off at low intensity. 1 mg/mL protein was treated with Turbo DNase I (Ambion) and high (2.5 U/mL) or low (0.8 U/mL) RNase I (Ambion). Dynabeads protein G (Invitrogen) were resuspended in lysis buffer containing 5 µg YBX1 antibody (Abcam). Precleared lysate was added to the magnetic beads for immunoprecipitation at 4 °C O/N. RNA 3′ ends were dephosphorylated and an infrared 3′ RNA adapter was ligated. Protein-RNA complexes were isolated following electrophoresis on a 4–12% NuPAGE Bis-Tris gel (Invitrogen). Precipitated RNA was reverse transcribed in RNA/primer mix containing different Rclip primers with individual barcode sequences for each replicate. cDNA was purified via AMPure XP bead capture (Beckman Coulter) and circularised using CircLigase™ II ssDNA Ligase (Cambio). Circularised cDNA was purified via AMPure XP bead capture (Beckman Coulter) and PCR amplification was carried out using Platinum™ II hot-start PCR master mix (2X) (Invitrogen) and primer mix P5Solexa/P3Solexa, 10 µM each at 19 cycles. The cDNA library was run on a 6% TBE gel (Invitrogen). cDNA was size selected between 145 and 400 nt and gel purified until primer dimers were no longer present [39]. Three independent biological replicates were prepared for sequencing on the NextSeq 500 (Illumina). Bioinformatics was performed on the web based iCount software (http://icount.biolab.si/). Mapping of YBX1 cross-link sites to regions of respective genes was visualised in the UCSC genome browser (http://genome.ucsc.edu/). iCLIP sequencing coverage and quality statistics for each sample are summarised in Suppl. File 3.

### 2.13 Mineralisation assay

Osteogenic medium was prepared fresh and comprised DMEM with 0.5% FBS, 1% penicillin/streptomycin, 10 nM dexamethasone and 50 µg/mL ascorbic acid. A monolayer with complete confluency was achieved 48 h after plating in 48 well plates. Wells were washed twice in PBS. Cells were treated with several CADD522 concentrations from 0.1 to 100 µM with equivalent DMSO concentration used as a control. The treatments were replenished every 2–3 d and for the last 48 h the medium was supplemented with 5 mM inorganic phosphates. The experiments ended 7 d after treatment at which point the cells were fixed in absolute ethanol overnight and stained with 40 mM Alizarin Red. The percentage area mineralised was quantified using ImageJ 1.53 K. Experiments were performed in quadruplicates in three independent experiments.

### 2.14 In vivo studies

The University of Sheffield Animal Welfare and Ethics Committee approved the animal experiments. Experiments were performed under licence in accordance with UK Home Office guidelines and under the Animals (Scientific Procedures) Act 1986. Six to seven-week-old female BALB/c nude mice (Charles River Laboratories) were acclimatised for one week prior to study. Mice were housed with a 12 h light–dark cycle at 22 °C and had free access to 2018 Teklad global 18% protein rodent diet containing 1.01% calcium (Harlan) and water. Mice were anesthetised by isoflurane inhalation before implanting with 2.5 × 10^5^ 143B + GFP-luc (OS) cells or 5 × 10^5^ TC71 + GFP-luc (ES) cells in 20 µL PBS onto the proximal tibia. Mice were weight- and initial IVIS signal-matched and then randomly divided into treatment groups (n = 8 mice/group). CADD522 (#5221975, ChemBridge) was produced at > 90% purity confirmed by HPLC, combined with DMSO/PBS (5%/95% v/v) and sonicated until soluble. The formulation was then filtered using a 0.22 μM PVDF filter. CADD522 treatment commenced the day after tumours became palpable. Mice received vehicle control (DMSO/PBS) or CADD522 (25 mg/kg) by IP five times per week for a maximum of 7 weeks. Tumours were measured by callipers throughout the study twice per week blinded. Two perpendicular measurements were taken and volume was calculated by V = 0.523 × L × (S)^2^ where L and S refer to the largest and smallest measurement. At the end of the study, *ex vivo* imaging of internal organs was performed to detect metastasis. Prior to killing, mice were injected subcutaneously with 150 mg/kg XenoLight D-Luciferin substrate (Perkin Elmer) that was left to disperse for 5 min. Dissected organs were then imaged using the IVIS Lumina II. Legs and lungs were dissected and fixed in 10% formalin.

### 2.15 Micro-CT

Fixed tibiae were scanned using a SkyScan 1172 desktop micro-CT (Bruker) at 8 µm resolution with the X-ray source set to 50 kV, 200 uA and using a 0.5 mm aluminium filter. Images were captured every 0.7°. Scanned images were reconstructed using Skyscan NRecon v1.6.9 (Bruker). The region-of-interest (ROI) for the total bone volume was selected to include both the tibia and fibula and was determined at the top of the bone as soon as the tibia enters the image to the lower point where the tibia and fibula meet.

### 2.16 Histology

Bones were fixed in neutral buffered formalin for 48 h after which they were transferred to 70% ethanol. Bones were then decalcified in 10% EDTA, embedded in paraffin and 5 µm sections produced. Sections were dewaxed in xylene, rehydrated through graded alcohols and stained in haematoxylin (VWR) and eosin. The sections were dehydrated through graded alcohols and coverslips were mounted in DPX and imaged using an Olympus BX51 (Olympus Life Sciences) and Pannoramic 250 Flash III slide scanner (3DHISTECH).

### 2.17 Statistics

Variability between sequencing libraries was evaluated using scatter plots, replicate-to-replicate DE size split box plots, intersection and Jaccard similarity analysis (40). Empirical DE analysis was confirmed by parametric (t-tests) and non-parametric (Mann-Whitney-U) tests. For statistical tests we considered p < 0.05 as significant. All sequencing data presented in this study fulfilled log_2_ fold change > 1, p < 0.05 and false discovery rate ≤ 5% criteria. Proliferation and mineralisation data was tested for normality and analysed in Prism (v9) (GraphPad) with p < 0.05 considered significant. A one-way ANOVA was used to identify any differences in proliferation across the concentration range tested compared to the untreated control. A post-hoc Dunnett’s multiple comparison was then used to determine which concentration impacted proliferation compared to the untreated control. Analysis was performed for each independent experiment and for the average results across experiments for each cell line and exposure period. Prism (v9) (GraphPad) was also used to generate the survival curves, with a Log-rank (Mantel-Cox) test used to determine if the survival rates were different between groups. Metastasis-free survival data was generated using the length of time before the mouse had to be killed due to metastasis-associated adverse effects. Overall survival data was generated using the length of time before the mouse had to be killed due to adverse effects irrespective of metastasis.

## 3 Results

### 3.1 HD adapters reveal novel sRNA species in CS

**Fig. 1:**
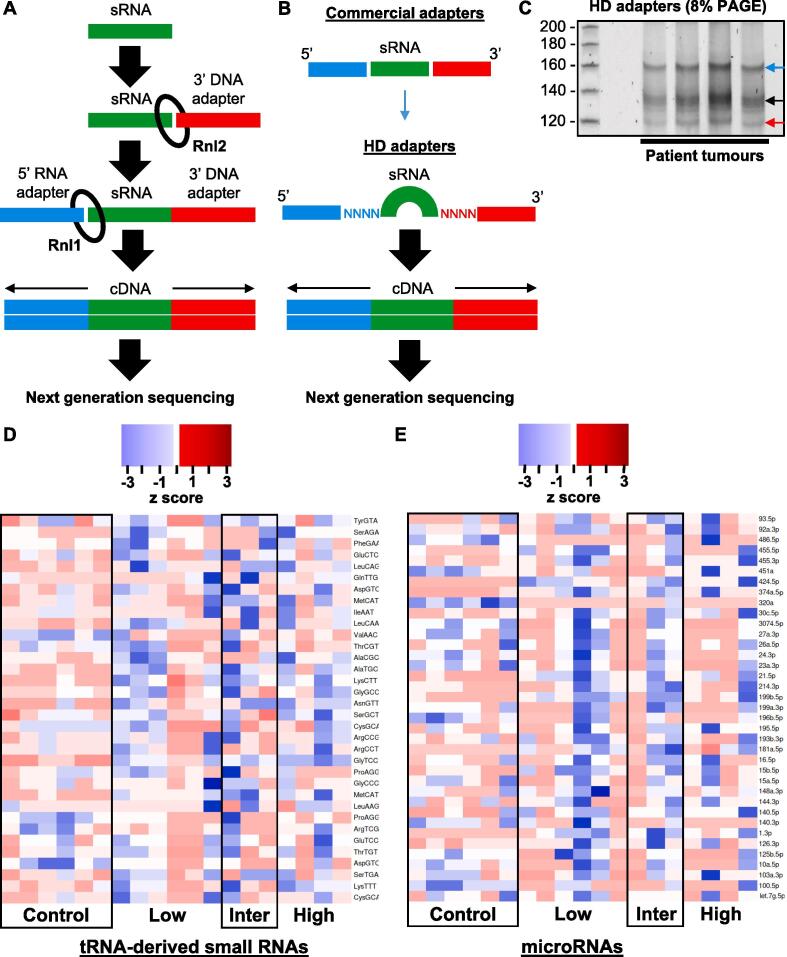
sRNA-seq schematic, 8% PAGE and heat map-based normalised sRNA expression hierarchical linear scale. A. Traditional sRNA-seq employs Rnl2 to ligate a 3′ DNA adapter and Rnl1 to ligate a 5′ RNA adapter to an sRNA sample. The product is then converted to cDNA before undergoing next generation sequencing. B. Commercial adapters are a fixed sequence. Rnl2 and Rnl1 are biased for ligating adapters and sRNAs where there is a good complementarity between the sequences or annealing efficiency. sRNAs that do not have a good annealing efficiency are not ligated to adapters and are not sequenced. HD adapters contain four random nucleotides on the ligating ends of commercial adapters creating a 2^8^ sequence pool that increases the annealing efficiency between sRNAs and adapters. C. 8% PAGE as part of the size selection step. Upper band (blue arrow) revealed tRF and ysRNA libraries. Middle band (black arrow) were miRNAs. Adapter dimers (red arrow) were omitted to increase depth for tRF and miRNA sequencing. D. Statistically significant tRF expression comparisons between control, low-grade, intermediate-grade and high-grade clinical CS samples. E. Statistically significant miRNA expression comparisons between control, low-grade, intermediate-grade and high-grade clinical CS samples. Z-score refers to high (red) and low (blue) sRNA expression using normalised values when compared with the mean of total sequencing reads.

**Fig. 2:**
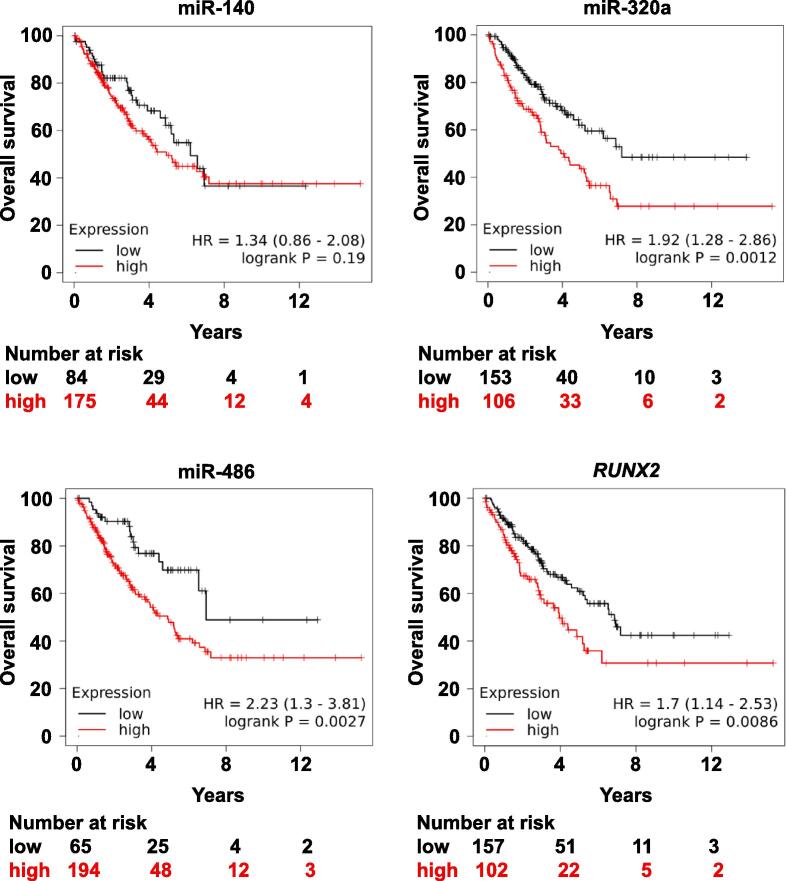
Mir-140, mir-320a, mir-486 and *RUNX2* expression is associated with lower sarcoma patient overall survival. The relationship between selected DE transcripts and sarcoma patient overall survival was assessed by Kaplan–Meier plotter (https://kmplot.com/analysis), which includes gene (mRNA, miRNA) and protein expression and survival data in over 30,000 samples from 21 tumour types. Samples were divided into two groups with high (red) and low (black) expression. Hazard ratios (HR) and logrank p values are shown. miR-140 expression had a lower prognosis (HR, 1.34) but this was non-significant.

**Fig. 3:**
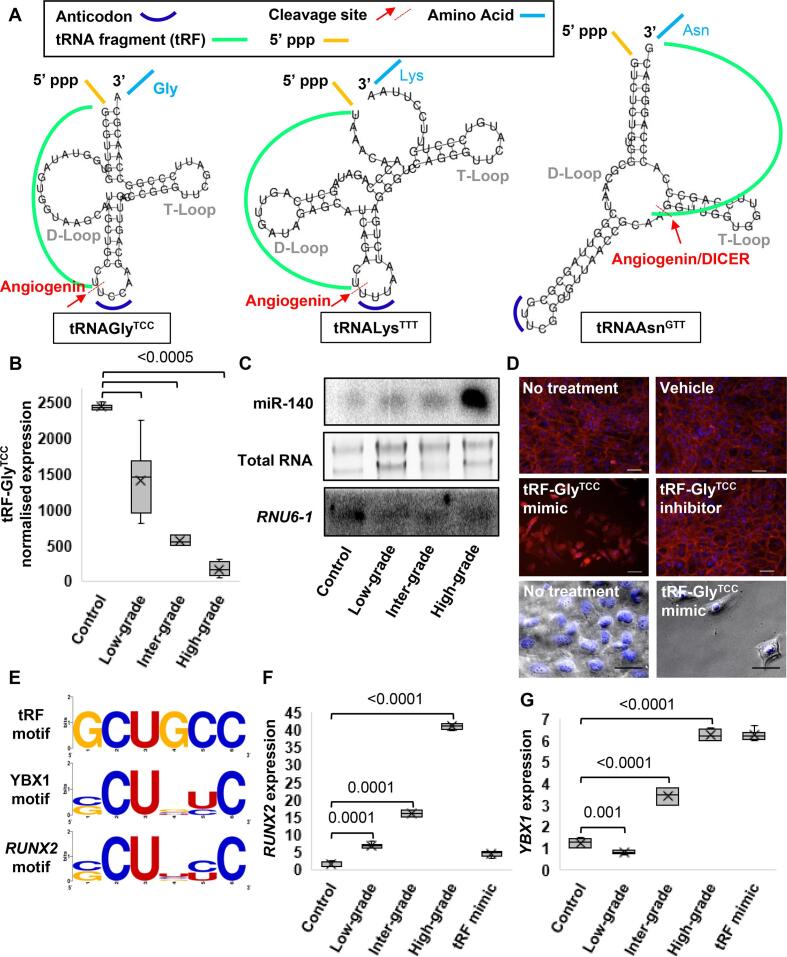
tRF-Gly^TCC^ loss-of-function is associated with CS progression. A. Full length tRNA secondary structures were generated using tRNAscan-SE [57] and Forna [58]. tRNAGly^TCC^ with a 5′ end cleavage between C and U. tRNALys^TTT^ with a 5′ end cleavage between U and U. tRNAAsn^GTT^ with a 3′ end cleavage between A and G. B. Normalised count read matrix represented as a box and whisker plot for tRF-Gly^TCC^ expression between tissue/tumour grades. C. Northern blot for miR-140 using an antisense 3′ end probe in control, low-grade, intermediate-grade and high-grade clinical samples. RNU6-1 and total RNA show equal (but low) loading to the membrane. D. CS micromass’ stained with DAPI to show nuclear DNA and Texas Red-X Phalloidin to show F-actin. Photomicrographs were taken at the most abundant cellular region of each micromass. Scale bars are 100 µm. E. Sequence logo analysis for tRF-Gly^TCC^, YBX1 and RUNX2. F. Using stored RNA from sequencing experiments and RNA isolated from micromass experiments, ectopic tRF-Gly^TCC^ reduced RUNX2 mRNA and increased G. YBX1 mRNA. Data was normalised to ACTB expression. Statistical significance was calculated using an unpaired *t*-test.

We collected CS patient tumour samples (low, intermediate and high-grade) and used HD adapters to generate sRNA-derived cDNA libraries (Fig. 1A, B). There was an unexpected sRNA band ∼ 20 nt above the miRNA band at the size selection step of the protocol (Fig. 1C). We isolated this unexpected band with the miRNAs and next generation sequencing revealed reads mapping to miRNAs, tRFs and ysRNAs (Suppl. File 1). Bioinformatics revealed significant DE in 34 tRFs (Fig. 1D) and 37 miRNAs (Fig. 1E) when CS samples were compared to controls. We selected miR-140, miR-320a and miR-486 because of their relevance to sarcoma patient overall survival (Fig. 2) and tRF-Gly^TCC^, tRF-Lys^TTT^, tRF-Asn^GTT^ (Fig. 3A) because they had the highest DE in our samples for downstream analysis [21], [41], [42]. An observation in our dataset was that tRF-Gly^TCC^ displayed a significant lineal attenuation as CS progressed (Fig. 3B). MiR-140 showed significant upregulation in high-grade tumours (Fig. 3C; Supplementary Fig. S1). We noted from our previous work in embryonic bone development that miR-140 is a *RUNX2* master regulator during skeletogenesis and mesenchymal cell migration [43]. We also observed that *RUNX2* expression was associated with lower sarcoma patient overall survival (HR, 1.7; 95% CI, 1.14–2.53; p = 0.0086) (Fig. 2).

### 3.2 tRF-Gly^TCC^ gain-of-function dissociates CS micromass *in vitro*

Using the datasets generated from the hypothesis-free sequencing approach, we next performed *in vitro* loss- and gain-of-function experiments for our candidate sRNAs. Our model system was a 3D micromass culture, where bone/cartilage micromass’ reflect the natural tissue state better than conventional 2D cell culture [44]. We used intermediate-grade human CS cells for the micromass model so that we could better quantify effects in both directions, with tumour suppressive effects likely to display low-grade phenotypes and oncogenic effects likely to display high-grade phenotypes. We induced miR-140, miR-320a, miR-486, tRF-Gly^TCC^, tRF-Lys^TTT^ and tRF-Asn^GTT^ gain- and loss-of-function. Nuclear and F-actin staining showed no obvious phenotypic response except for the tRF-Gly^TCC^ mimic (Fig. 3D; Suppl. Fig. 1). Though each candidate mimic and inhibitor may have affected gene expression, the tRF-Gly^TCC^ gain-of-function presented low cell number, shrunken nuclei and cell morphology changes (Fig. 3D). The most quantifiable change was nuclear DNA condensation and decrease in nuclear size shown by fluorescent DNA staining (Suppl. Fig. 1). We detected > two-fold decrease in cell nuclear area when treated with a tRF-Gly^TCC^ mimic (215 ± 16.8 µm^2^; n = 47 nuclei) when compared to untreated cells (488 ± 12.4 µm^2^; n = 230 nuclei) (Fig. 3D; Suppl. Fig. 1).

### 3.3 Conserved sequence motif in tRF-Gly^TCC^, YBX1 and *RUNX2*

**Fig. 4:**
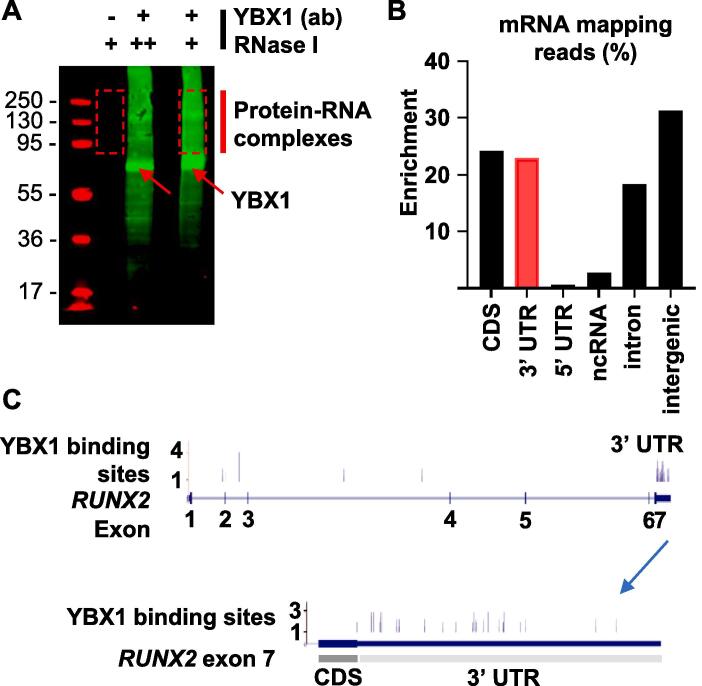
YBX1 physically interacts with *RUNX2* 3′ UTR. A. PAGE gel showing YBX1 pull down. B. mRNA-seq reveals mRNA regions bound to by YBX1. C. YBX1 physically interacts with several regions across the *RUNX2* mRNA with marked enrichment in the 3′ UTR.

tRF-Gly^TCC^ was now our lead candidate sRNA with a role in CS progression. A previous study reported that tRF-Gly^TCC^ is a key player in AGO2-independent RNA silencing by sequestering YBX1 and inhibiting YBX1-mediated stabilisation of pro-metastatic mRNAs in breast cancer [24]. We performed sequence logo analysis for tRF-Gly^TCC^ and the YBX1 nucleic acid recognition sequence confirming that these molecules share a conserved (C/G)CU(C/G/U)(C/U)C sequence motif (Fig. 3E). Together with our sequencing data that showed an inverse correlation between tRF-Gly^TCC^ and miR-140 expression (Fig. 3B, 3C) and from our previous work that miR-140 is an embryonic driver for *RUNX2* [43], we included *RUNX2* in the sequence logo analysis. The *RUNX2* 3′ UTR contained several (C/G)CU(C/G/U)(C/U)C motifs (Fig. 3E). Using stored RNA from the sequencing experiments and RNA extracted from the gain- and loss-of-function experiments, we performed qPCR and detected a significant increase in *RUNX2* expression in CS samples (Fig. 3F) that were inverse to tRF-Gly^TCC^ expression (Fig. 3B). These experiments supported a model where tRF-Gly^TCC^ suppresses CS progression by downregulating *RUNX2* translation; by YBX1 displacement according to the mechanism of action revealed in breast cancer [24]. Further supporting this model was that *RUNX2* mRNA was attenuated in micromass cultures exposed to tRF-Gly^TCC^ gain-of-function (Fig. 3F) whilst *YBX1* mRNA was upregulated in response to the mimic (Fig. 3G). We developed an individual nucleotide resolution cross-linking and immunoprecipitation (iCLIP) method for CS cells and these experiments confirmed that endogenous YBX1 (Fig. 4A) physically interacts with *RUNX2* mRNA (Fig. 4B) with significant enrichment in the 3′ UTR (Fig. 4C).

### 3.4 RUNX2/ATP synthase inhibitor CADD522 as a single agent reduces PBC progression, PBC-induced bone disease and increases metastasis-free survival *in vivo*

**Fig. 5:**
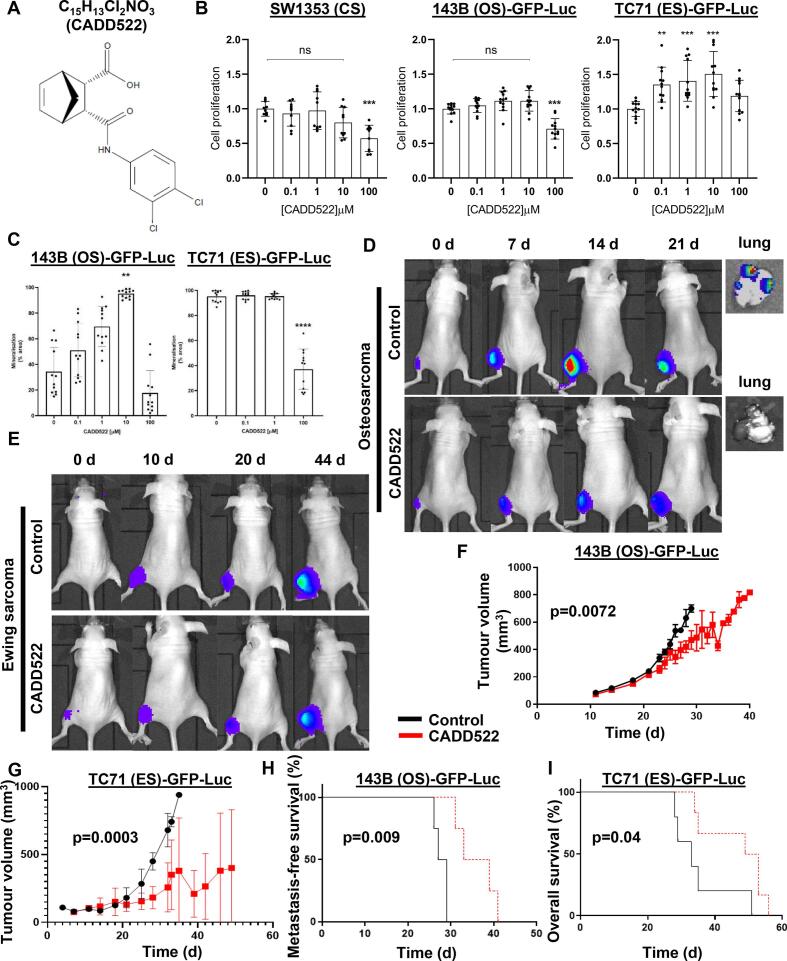
CADD522 significantly increases OS metastasis-free survival and ES overall survival. A. C_15_H_13_Cl_2_NO_3_ chemical structure. B. CS, OS and ES cell (GFP-luciferase tagged for the animal experiments) viability and proliferation in response to CADD522 *in vitro* after 72 h. ***p < 0.005, **p < 0.01. Each dot is one replicate. Four replicates were tested in three independent experiments. C. OS and ES mineralisation measured over 7 d treated with a CADD522 dose range. Data shown is mean +/- SD from three independent experiments, four wells per experiment. Plot shows a one-way ANOVA on the mean of the independent experiments with Dunnett’s multiple comparison (n = 3). ****p < 0.001, **p < 0.01. D. Tumour luminescence in CADD522 treated OS animals. Luminescence decreases (21 d) after a spike (14 d) in the control mice because OS tumours are centrally necrotic, but the tumour volume still increases. E. Tumour luminescence in CADD522 treated ES animals. F. Tumour volumes in OS animals when treated with CADD522 versus non-treated controls. G. Tumour volumes in ES animals when treated with CADD522 versus non-treated controls. H. Metastasis-free survival in OS animals when treated with CADD522 versus non-treated controls. I. Overall survival in ES animals when treated with CADD522 versus non-treated controls. No metastases were detected in the ES model.

Our experimental data combined with some hypotheses suggested that increased *RUNX2* expression enhanced CS progression, but RUNX2 translation was normally held in check by tRF-Gly^TCC^. To determine if RUNX2 pharmacological regulation would prove to be a potential therapeutic option in PBC, we synthesised the novel compound computer aided drug design molecule 522 (CADD522) (Fig. 5A). We revisited our early hypothesis that CS datasets may steer future OS and ES experiments/treatments, so we assessed CADD522 effect on CS, OS and ES cells *in vitro*. CS and OS cells showed significantly reduced cell proliferation after 100 µM CADD522 treatment for 72 h whilst the effect on ES cell proliferation showed a bimodal effect with lower doses increasing proliferation (Fig. 5B). To further explore the lower CADD522 dose effect on PBC cell function we examined mineralisation. CS cells failed to mineralise *in vitro* but both OS and ES cells produced a mineralised matrix. CADD522 increased OS cell mineralisation in a dose-dependent manner up to 10 µM but had no effect on ES cell mineralisation at these lower concentrations (Fig. 5C). CADD522 at 100 µM significantly reduced mineralisation in both cell lines probably due to its effect on cell viability even in these non-proliferative conditions. CADD522 was then assessed *in vivo* using OS and ES xenograft mouse models; a high-grade CS xenograft has not yet been developed and we reasoned that a subcutaneous model would not accurately reflect the disease. CADD522 significantly reduced tumour luminescence in both models (Fig. 5D, E) and significantly reduced tumour volumes in OS (p = 0.0072) and ES (p = 0.003) (Fig. 5F, G). CADD522 showed significantly increased metastasis-free survival in OS (p = 0.0091) and significantly increased overall survival in ES (p = 0.043) (Fig. 5H, I).

**Fig. 6:**
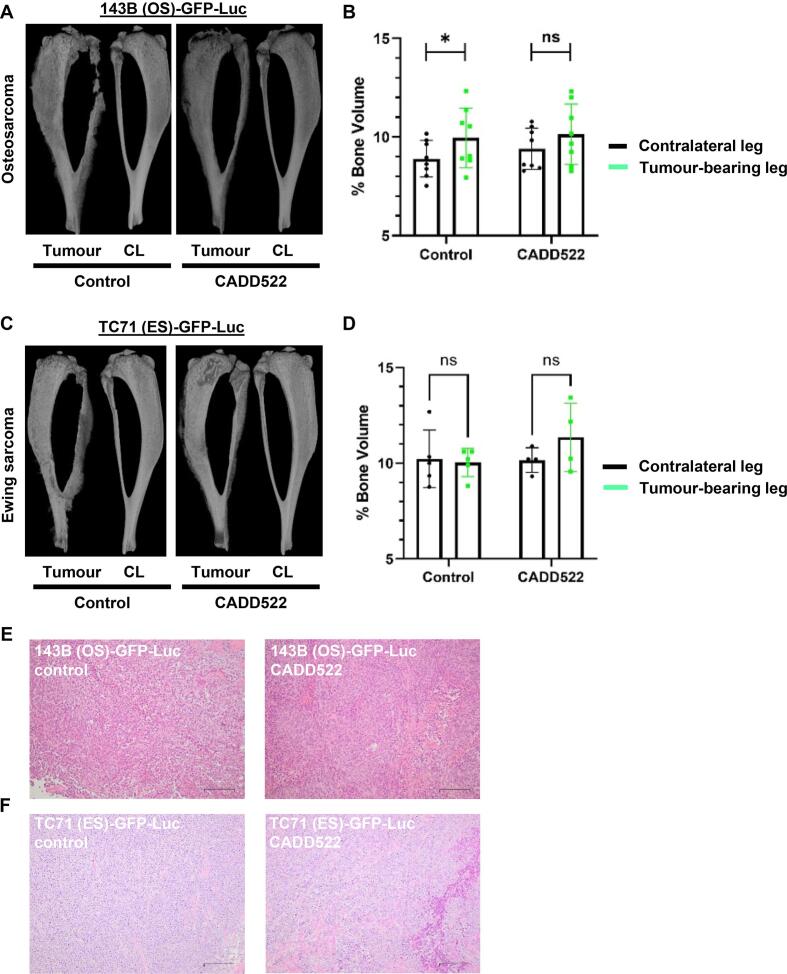
CADD522 reduces PBC-induced bone disease. A. OS micro-CT image showing PBC-induced bone disease (severe mixed effects of ectopic bone formation and lytic bone destruction). B. Bone volume quantification and comparison to the non-tumour bearing contralateral (CL) leg. *p < 0.05. C. ES micro-CT image showing a similar architectural phenotype to OS. D. Bone volume quantification and comparison to the non-tumour bearing contralateral (CL) leg. E. Representative OS tumour H&E sections. F. Representative ES tumour H&E sections. Scale bars are 50 µm.

PBC clinical complications can include PBC-induced bone disease that manifests as ectopic osteoid/cartilage matrix deposition and/or lytic destruction. CADD522 effect on bone disease observed in these models was assessed by micro-CT. When comparing OS tumour effect on bone volume, control mice presented with severe mixed effects including ectopic bone formation and lytic bone destruction (Fig. 6A). OS tumours caused significantly increased bone volume in the tumour bearing leg when compared to the non-tumour bearing contralateral leg (p = 0.02) (Fig. 6B). In CADD522 treated mice the bone volume difference was no longer statistically significant suggesting less severe bone disease (Fig. 6B). ES tumours demonstrated a similar phenotype with visible ectopic bone formation and lytic destruction (Fig. 6C), but the bone volume data did not reach statistical significance (Fig. 6D). Tumour histology at endpoint showed that OS tumours obtained from mice treated with CADD522 were more densely packed, generally more vascularised and organised (arranged in chords) when compared to non-treated controls (Fig. 6E). ES tumours showed a similar pattern of being generally more vascularised when treated with CADD522 with both groups showing a high level of central necrosis likely due to the prolonged experiment duration with this model (Fig. 6F).

## 4 Discussion

sRNA-seq studies are biased towards some sRNA sequences because commercially available library kits employ adapters with a fixed sequence [25], [26], [27]. If the adapters have a low complementarity to sRNAs in a sample, T4 RNA ligase enzymes disregard those sRNAs that are then not included in the library prior to sequencing. We previously developed HD adapters that contain four randomly assigned nucleotides at the ligating ends of TruSeq adapters providing a pool of 2^8^ sequences for each ligating end, which increased the annealing efficiency between sRNAs and adapters [28], [29]. HD adapters significantly increased low abundance sRNA representation and allowed for new sRNAs to be identified [29]. Here, we used HD adapters to characterise the sRNA landscape in CS patient tumours (low- to high-grade). As well as several miRNAs, we detected several tRFs that were DE in CS progression. We observed that tRF-Gly^TCC^ was gradually reduced in CS progression and miR-140, known to promote *RUNX2* expression by *HDAC4* silencing [43], increased in CS progression. Sequence logo analysis showed that tRF-Gly^TCC^ and *RUNX2* shared a (C/G)CU(C/G/U)(C/U)C sequence motif in their 3′ ends. This motif was reported in breast cancer to be a YBX1 recognition sequence [24]. YBX1 is known to stabilise pro-metastatic transcripts [24]. We developed an iCLIP protocol for our PBC model to empirically demonstrate that YBX1 physically interacts with *RUNX2*, which showed significant enrichment in the 3′ UTR. We hypothesised that comparable to the breast cancer model, tRF-Gly^TCC^ interacted with YBX1 to blunt a potential oncogenic protein-RNA interaction, though it was undetermined whether this interaction caused apoptosis or reduced cell viability. In our PBC model we have shown that tRF-Gly^TCC^ is gradually deactivated and that *RUNX2* mRNA is a YBX1 protein target. The discovery of this YBX1/tRF-Gly^TCC^/*RUNX2* interaction led to the development and testing of the RUNX2 transcription factor and ATP synthase inhibitor CADD522 [45], [46] as a novel single agent PBC treatment without surgery *in vivo*. Animals implanted with human PBC and then treated with CADD522 showed significantly reduced tumour luminescence, tumour volumes, increased metastasis-free and overall survival and reduced PBC-induced bone disease.

Aside from tRF-Gly^TCC^ and *RUNX2* as PBC components, we identified YBX1 as the master regulator mediating these molecules’ effects on PBC progression. YBX1 is one of the most overexpressed oncogenes observed in human cancer and is highly heterogeneous with several variants and post-translational modifications [47], [48]. YBX1 was previously identified as a critical regulator of *HIF1A* expression in sarcoma leading to enhanced metastatic capacity *in vivo* [49]. A number of YBX1 target transcripts encode metastatic drivers including *EIF4G1*, *ITGB4*, *AKT1* and *ADAM8* [24]. *RUNX2* sequence analysis, where *RUNX2* was positively expressed when compared to tRF-Gly^TCC^ negative expression, revealed several YBX1 recognition motifs in the protein-coding sequence and 3′ UTR. Transfecting a tRF-Gly^TCC^ mimic reduced *RUNX2* expression to control levels. iCLIP sequencing showed that YBX1 physically interacts with *RUNX2* demonstrating a new target for YBX1 as well as a novel mechanism for PBC progression. YBX1 is colloquially regarded as ‘undruggable’, but the data presented here shows potential for targeting YBX1-interacting sRNAs and mRNAs such as *RUNX2* instead of YBX1 directly. Although this approach was not the sole focus of this study, as a tRF-Gly^TCC^ mimic could have been used *in vivo*, this tactic in future studies may show positive outcomes.

We searched for computational studies reporting potential binding sites for small molecules that interact with the RUNX2 transcription factor DNA binding site [50]. RUNX2 does not contain a well-defined binding site that typically occurs with other macromolecules such as enzymes. To identify likely DNA interacting site compounds, a previous study had obtained the RUNX2 solvent-accessible surface [51]. A hypothetical sphere set was generated that filled probable binding sites on the solvent-accessible surface using the sphgen programme and DOCK method [52]. These studies produced the identification and experimentation of C_15_H_13_Cl_2_NO_3_ or computer aided drug design molecule 522 (CADD522). CADD522 negatively regulated RUNX2 target gene transcription including *MMP13*, *VEGF* and *SLC2A1* in breast cancer cells [45]. Treatment with CADD522 in MMTV-PyMT mice resulted in tumour incidence delay, reduction in tumour burden and a decrease of tumour volume with no apparent toxicity [45]. A follow-up study showed that CADD522 also inhibited mitochondrial oxidative phosphorylation by decreasing the oxygen consumption rate plus ATP production in human breast cancer cells in a RUNX2-independent manner [46]. CADD522 preclinical assessment demonstrated effects on PBC models both *in vitro* and *in vivo*. CADD522 proved to be cytotoxic to CS and OS cells *in vitro* and PBC xenograft mouse models treated with the compound demonstrated significantly reduced tumour size, increased metastasis-free and overall survival as well as reduced PBC-induced bone disease. We observed no toxicity in the animals when treated with 25 mg/kg over the study period. Dedicated independent pharmacokinetic and pharmacodynamic studies are being performed. H&E staining showed CADD522 increased tumour vascularity meaning somewhat improved oxygenation and reduced hypoxia. Intra-tumour hypoxia has previously been shown to accelerate metastasis by increasing circulating tumour cell (CTC) clustering [53]; VEGF targeting led to primary tumour shrinkage, but increased intra-tumor hypoxia resulting in a higher CTC cluster shedding rate and metastasis formation [53]. Pro-angiogenic treatment increased primary tumour size, but suppressed CTC cluster formation and metastasis [53].

From the cell types affected and the treatments employed, childhood cancer (mostly blood, brain/spinal, sarcoma) is mechanistically dissimilar to adult cancer. The latter and more common cancers usually arise from decades of mutagenesis where their genomes are littered with single nucleotide variants (SNVs) and indels [54], [55]. Childhood cancer drivers mostly comprise complex structural abnormalities such as structural variants (SVs) and copy number variants (CNVs). SVs and CNVs may be the final step in overt transformation as emerging evidence suggests that childhood cancer may begin as an error during embryonic development [6], [7], [12]. Some childhood cancers will not be preventable meaning the best strategy to tackle these diseases is early and accurate diagnosis followed by gentle and effective treatment. OS and ES are high-grade at diagnosis [2] making it difficult to elucidate pro-metastatic events that occur immediately or soon after driver events but before tumour evolution has significantly progressed and made it almost impossible to untangle drivers and passengers. One approach was to use the more indolent CS subtype for proof-of-concept as a model system to generate data where there might be knowledge applicable to OS and ES. Here, tRF-Gly^TCC^ would not have been detected or characterised in OS or ES because it was depleted in high-grade samples. Discovery and characterisation of these novel molecular abnormalities, particularly a full profile of tRF-Gly^TCC^ by performing experiments that were not conducted here (e.g. cell viability, CRISPR knockouts, pull-downs, etc.), will provide new mechanistic understanding and may lead to new clinical targets and therapies [56].

## 5 Authors’ contributions

Study conception and design: D.G. and W.D.F. Experiments: D.G., V.L.T., L.T., K.M.S., C.S., N.W. and P.T. Bioinformatics: A.S. Data analysis and interpretation: D.G., A.S., D.J.E., A.G., T.D and W.D.F. Samples and pathology: D.G., S.C., L.J., V.S and I.M. Wrote the manuscript: D.G. Revised and approved the final manuscript: all authors.

## Declaration of Competing Interest

The authors declare that they have no known competing financial interests or personal relationships that could have appeared to influence the work reported in this paper.
