# Supplementary material for: YBX1-interacting small RNAs and RUNX2 can be blocked in primary bone cancer using CADD522
Source: J Bone Oncol. 2023 Mar 5;39:100474. doi: 10.1016/j.jbo.2023.100474 (PMC10015236; doi:10.1016/j.jbo.2023.100474)
Supplement: Supplementary data 1 [file mmc1.pptx]

## Slide 1
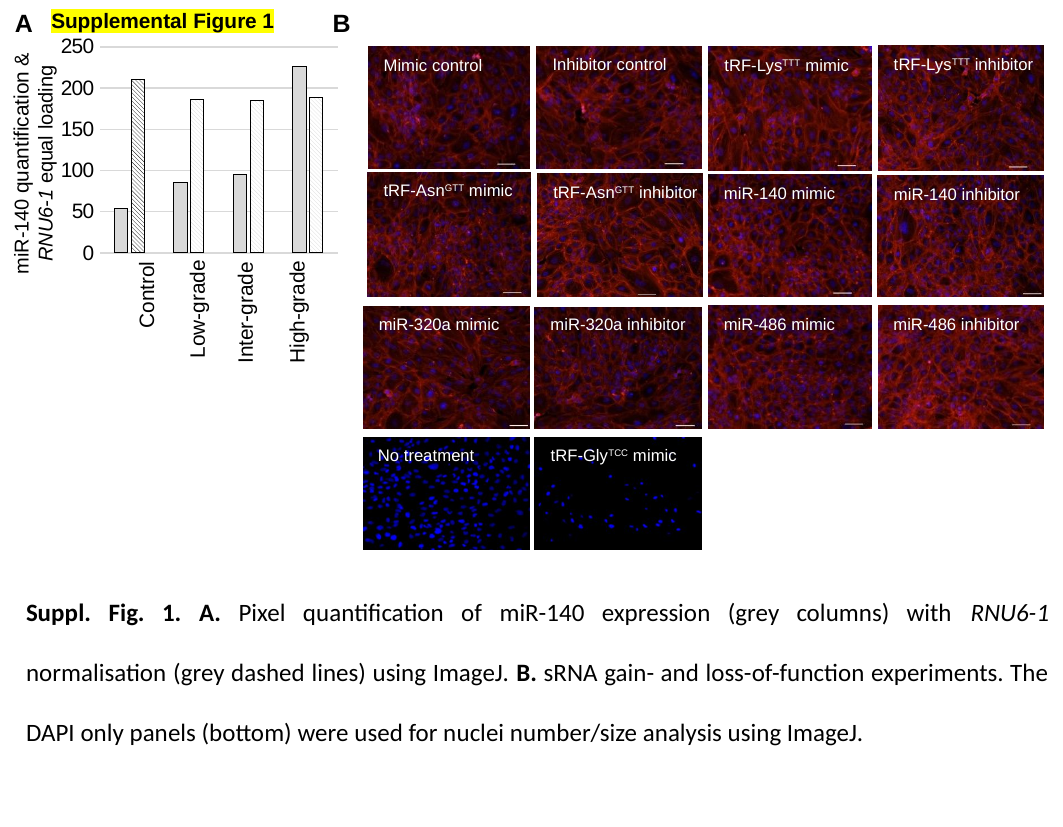

Supplemental Figure 1
A
B
### Chart
| Category | | |
|---|---|---|
| Control | 54.0 | 210.0 |
| Low | 85.0 | 186.0 |
| Intermediate | 95.0 | 185.0 |
| High | 226.0 | 188.0 |
tRF-LysTTT inhibitor
Inhibitor control
tRF-LysTTT mimic
Mimic control
miR-140 quantification & RNU6-1 equal loading
tRF-AsnGTT mimic
tRF-AsnGTT inhibitor
miR-140 mimic
miR-140 inhibitor
Control
Low-grade
High-grade
Inter-grade
miR-486 mimic
miR-486 inhibitor
miR-320a mimic
miR-320a inhibitor
tRF-GlyTCC mimic
No treatment
Suppl. Fig. 1. A. Pixel quantification of miR-140 expression (grey columns) with RNU6-1 normalisation (grey dashed lines) using ImageJ. B. sRNA gain- and loss-of-function experiments. The DAPI only panels (bottom) were used for nuclei number/size analysis using ImageJ.
