# Supplementary material for: YBX1-interacting small RNAs and RUNX2 can be blocked in primary bone cancer using CADD522
Source: J Bone Oncol. 2023 Mar 5;39:100474. doi: 10.1016/j.jbo.2023.100474 (PMC10015236; doi:10.1016/j.jbo.2023.100474)
Supplement: Supplementary data 2 [file mmc2.pdf]

Cell Line Authentication Report for Case Number C-26872

Statement

We have now completed DNA analysis of the samples presented for Cell Line Authentication

A DNA Profile has been generated from the sample provided by The University of Sheffield and compared to a profile located on the Cellosaurus Database. A Matching Percentage has been calculated and a value of 100% obtained. Please refer to the detailed results on the second page for further information and interpretation.

Analysis has been conducted using 8 loci allowing identical matches an approximate power of discrimination of 1 in 1,000,000,000. Please note that some cell lines may exhibit genetic instability as they proliferate leading to discrepancies within the DNA profiles examined.

Details of the DNA analysis as tested by PowerPlex® Fusion are shown on the next page.

11/10/2022

This report shall not be reproduced, except in full, without written approval of the laboratory. The validity of these results depends on the quality of the sample provided and the identification of samples being correct.

northgene™
A BIOFORTUNA brand

UKAS TESTING 7816

Laboratory Report

Test Requested	Cell Line Authentication
Case Number	C-26872
Date Sample Received	05/10/2022
Date Sample Tested	05/10/2022
Date Sample Reported	07/10/2022

Sample Name	Sample/Comparison Profile Source	Sample Number	DNA Number
SW1353+GFP-LUC	The University of Sheffield	S-1061347	D-1061347
SW1353	Cellosaurus Database	N/A	N/A

Table of Allelic Data

	Genotypes		
STR Locus	SW1353+GFP-LUC (Test Sample)	SW1353 (Comparison Sample)	Match vs. Mis-Match
D5	10 | 11	10 | 11	Match
D13	12 | 13	12 | 13	Match
D7	9 | 11	9 | 11	Match
D16	11 | 12	11 | 12	Match
vWA	16 | 17	16 | 17	Match
Amel	X | X	X | X	Match
TPOX	8 | 11	8 | 11	Match
CSF1PO	12 | 12	12 | 12	Match
THO1	6 | 9	6 | 9	Match

Matching Percentage: **100%**
Outcome: **Related**

The outcome percentage is calculated using a formulae which compares the number of alleles present against the number of alleles shared between the two DNA profiles. The outcome is designated one of the following statements based upon the outcome percentage:

Related (>80%)	The Cell Lines are considered to be related.
Inconclusive (56-79%)	Further profiling is required to determine whether the profiles are related.
No Match (55%>)	It is considered that the two cell lines are unrelated.
Misidentified	Cell Lines have been found to match a different donor within the database

Reported By: Ms. Ish Jones (Laboratory Scientist) [signature]

Authorised By: Mr. Benjamin Hickey (Laboratory Scientist) [signature]

Date: 11/10/2022

These test results should only be used in conjunction with a client's information. The results relate only to the items sampled, as received and tested at this time.

Amended Report: C-26696d

Cell Line Authentication Report for Case Number C-26696a

Statement

We have now completed DNA analysis of the samples presented for Cell Line Authentication

A DNA Profile has been generated from the sample provided by The University of Sheffield and compared to a profile located on the Cellosaurus Database. A Matching Percentage has been calculated and a value of 100% obtained. Please refer to the detailed results on the second page for further information and interpretation.

Analysis has been conducted using 8 loci allowing identical matches an approximate power of discrimination of 1 in 1,000,000,000. Please note that some cell lines may exhibit genetic instability as they proliferate leading to discrepancies within the DNA profiles examined.

Details of the DNA analysis as tested by PowerPlex® Fusion are shown on the next page.

13/10/2022

This report supercedes report reference C-26696a issued on 13/09/2022 . The reason for amendment is: Full name of supplied cell line added at customer request

This report shall not be reproduced, except in full, without written approval of the laboratory. The validity of these results depends on the quality of the sample provided and the identification of samples being correct.

northgene™
A BIOFORTUNA brand

UKAS TESTING 7816

Laboratory Report

Test Requested Cell Line Authentication

Case Number C-26696d
Date Sample Received 06/09/2022
Date Sample Tested 06/09/2022
Date Sample Reported 12/10/2022

Sample Name	Sample/Comparison Profile Source	Sample Number	DNA Number
143B+GFP-LUC	The University of Sheffield	S-1060848	D-1060848
143B	Cellosaurus Database	N/A	N/A

Table of Allelic Data

	Genotypes		
STR Locus	143B+GFP-LUC (Test Sample)	143B (Comparison Sample)	Match vs. Mis-Match
D5	13 | 13	13 | 13	Match
D13	12 | 12	12 | 12	Match
D7	11 | 12	11 | 12	Match
D16	10 | 13	10 | 13	Match
vWA	18 | 18	18 | 18	Match
Amel	X | X	X | X	Match
TPOX	11 | 11	11 | 11	Match
CSF1PO	12 | 12	12 | 12	Match
THO1	6 | 6	6 | 6	Match

Matching Percentage: **100%**
Outcome: **Related**

The outcome percentage is calculated using a formulae which compares the number of alleles present against the number of alleles shared between the two DNA profiles. The outcome is designated one of the following statements based upon the outcome percentage:

Related (>80%) The Cell Lines are considered to be related.
Inconclusive (56-79%) Further profiling is required to determine whether the profiles are related.
No Match (55%>) It is considered that the two cell lines are unrelated.
Misidentified Cell Lines have been found to match a different donor within the database

Reported By: Ms. Georgina Fair (Laboratory Scientist)

Authorised By: Ms. Eleanor Ralston (Senior Scientist)

Date: 13/10/2022

These test results should only be used in conjunction with a client's information. The results relate only to the items sampled, as received and tested at this time.

Amended Report: C-26696e

Cell Line Authentication Report for Case Number C-26696b

Statement

We have now completed DNA analysis of the samples presented for Cell Line Authentication

A DNA Profile has been generated from the sample provided by The University of Sheffield and compared to a profile located on the Cellosaurus Database. A Matching Percentage has been calculated and a value of 97% obtained. Please refer to the detailed results on the second page for further information and interpretation.

Analysis has been conducted using 8 loci allowing identical matches an approximate power of discrimination of 1 in 1,000,000,000. Please note that some cell lines may exhibit genetic instability as they proliferate leading to discrepancies within the DNA profiles examined.

Details of the DNA analysis as tested by PowerPlex® Fusion are shown on the next page.

13/10/2022

This report supercedes report reference C-26696b issued on 13/09/2022 . The reason for amendment is: Full name of supplied cell line added at customer request

This report shall not be reproduced, except in full, without written approval of the laboratory. The validity of these results depends on the quality of the sample provided and the identification of samples being correct.

northgene™
A BIOFORTUNA brand

UKAS TESTING 7816

Laboratory Report

Test Requested	Cell Line Authentication
Case Number	C-26696e
Date Sample Received	06/09/2022
Date Sample Tested	06/09/2022
Date Sample Reported	12/10/2022

Sample Name	Sample/Comparison Profile Source	Sample Number	DNA Number
TC71+GFP-LUC Ewing sarcoma	The University of Sheffield	S-1060849	S-1060849
TC-71 CVCL_2213	Cellosaurus Database	N/A	N/A

Table of Allelic Data

	Genotypes		
STR Locus	TC71+GFP-LUC Ewing sarcoma (Test Sample)	TC-71 CVCL_2213 (Comparison Sample)	Match vs. Mis-Match
D5	10 | 10	10 | 10	Match
D13	11 | 12	11 | 12	Match
D7	10 | 10	10 | 10	Match
D16	11 | 14	11 | 14	Match
vWA	17 | 18	17 | 17	Mis-Match
Amel	X | Y	X | Y	Match
TPOX	8 | 9	8 | 9	Match
CSF1PO	10 | 11	10 | 11	Match
THO1	9.3 | 9.3	9.3 | 9.3	Match

Matching Percentage: **97%**
Outcome: **Related**

The outcome percentage is calculated using a formulae which compares the number of alleles present against the number of alleles shared between the two DNA profiles. The outcome is designated one of the following statements based upon the outcome percentage:

Related (>80%)	The Cell Lines are considered to be related.
Inconclusive (56-79%)	Further profiling is required to determine whether the profiles are related.
No Match (55%>)	It is considered that the two cell lines are unrelated.
Misidentified	Cell Lines have been found to match a different donor within the database

Reported By: Ms. Georgina Fair (Laboratory Scientist)

Authorised By: Ms. Eleanor Ralston (Senior Scientist)

Date: 13/10/2022

These test results should only be used in conjunction with a client's information. The results relate only to the items sampled, as received and tested at this time.

Sample File	Sample Name	SOS	SQ	SSPK	MIX	OMR	CGQ
Alleic_Ladder_2_H04_22.hid	Alleic_Ladder_2				NA	NA	

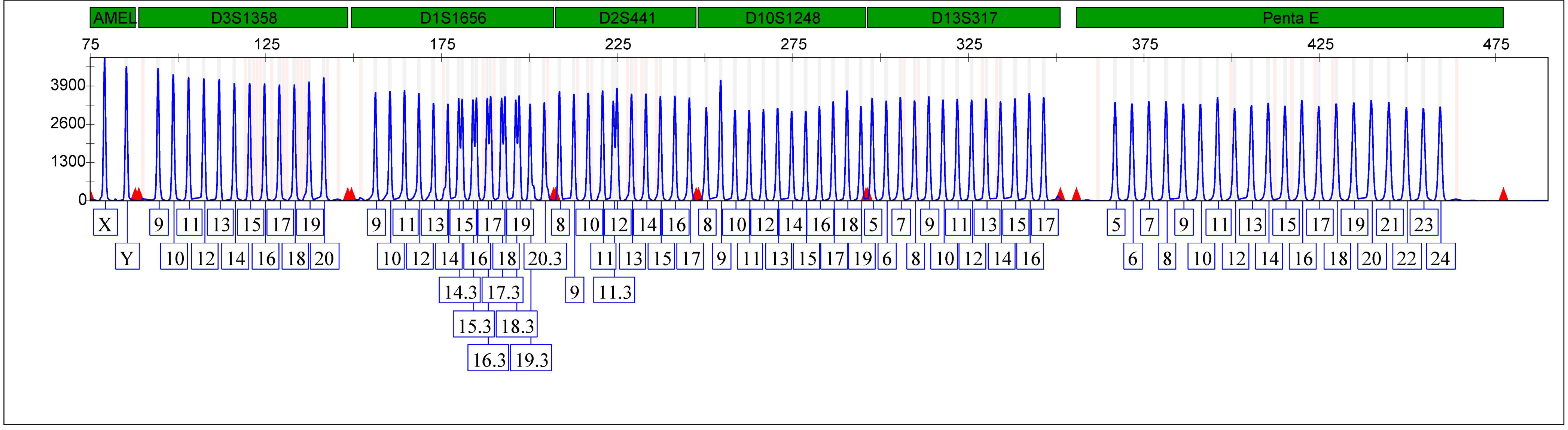

Sample File	Sample Name	SOS	SQ	SSPK	MIX	OMR	CGQ
Alleic_Ladder_2_H04_22.hid	Alleic_Ladder_2				NA	NA	

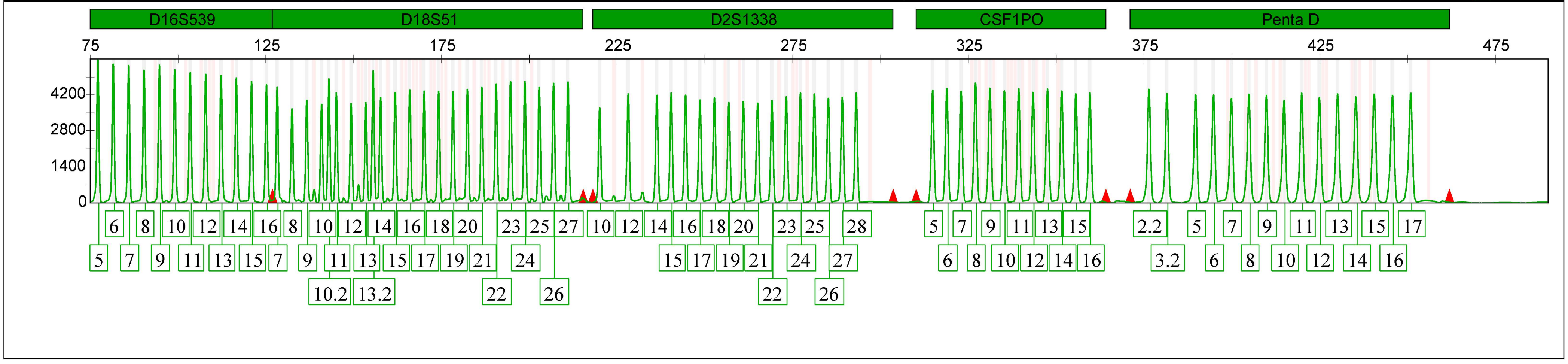

Sample File	Sample Name	SOS	SQ	SSPK	MIX	OMR	CGQ
Alleic_Ladder_2_H04_22.hid	Alleic_Ladder_2				NA	NA	

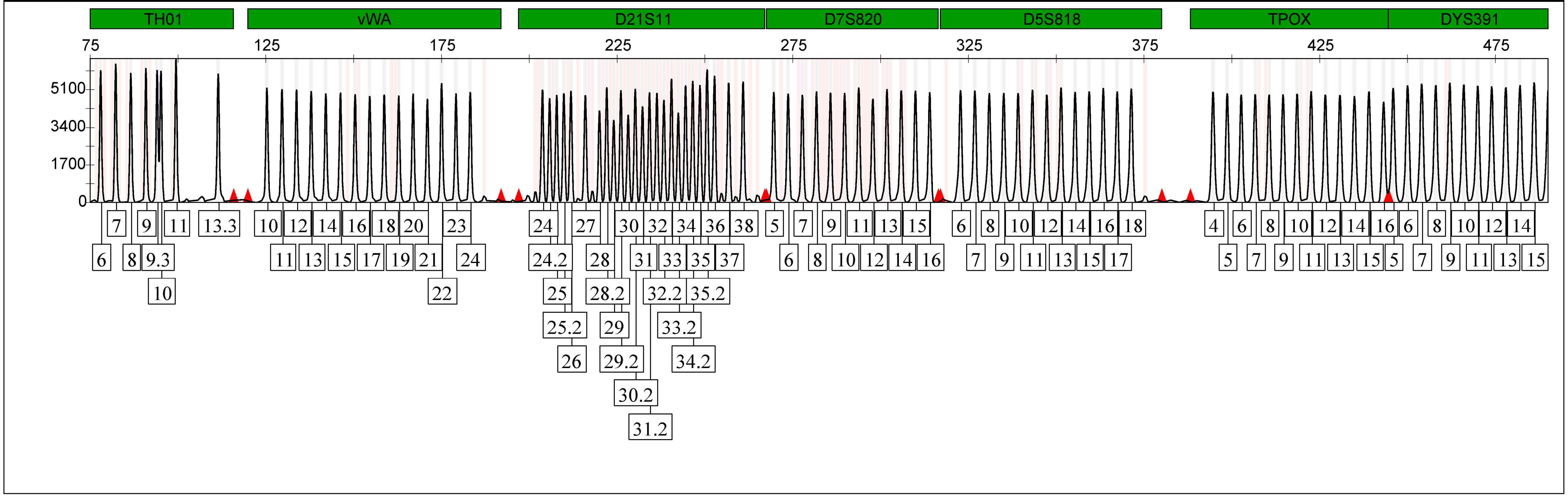

Sample File	Sample Name	SOS	SQ	SSPK	MIX	OMR	CGQ
Alleic_Ladder_2_H04_22.hid	Alleic_Ladder_2				NA	NA	

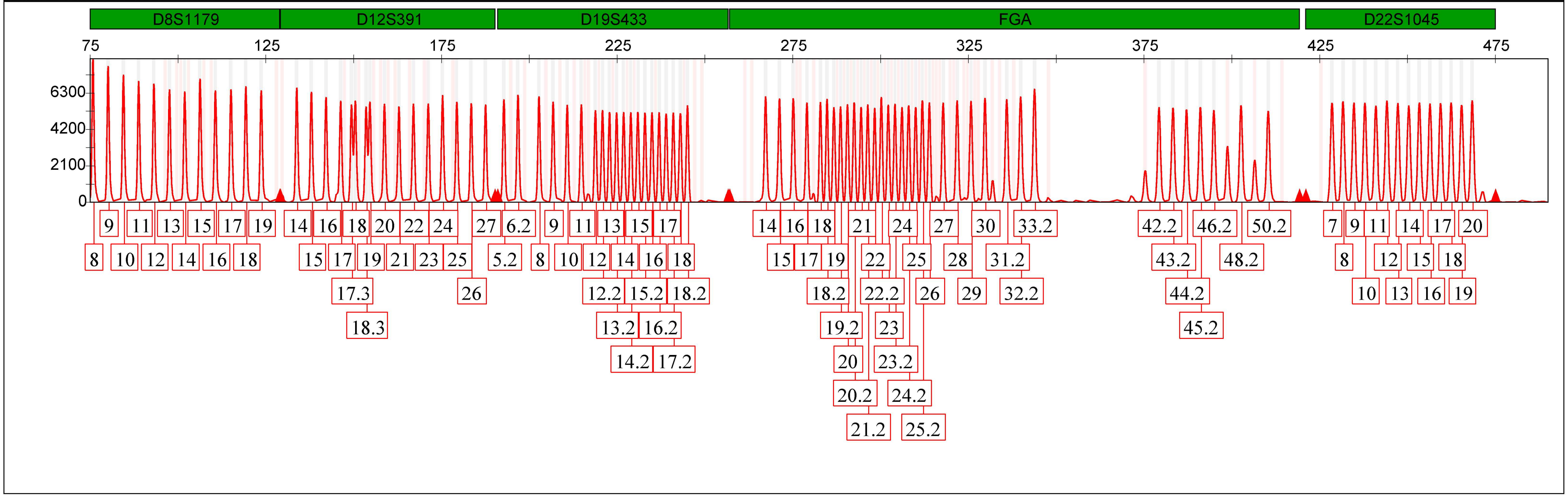

Applied Biosystems
GeneMapper® ID-X 1.5

Project: LW10100280+281-22

Sample File	Sample Name	SOS	SQ	SSPK	MIX	OMR	CGQ
D-1060848_Cell Line_C-26696_F03_18.hid	D-1060848_Cell Line_C-26696						

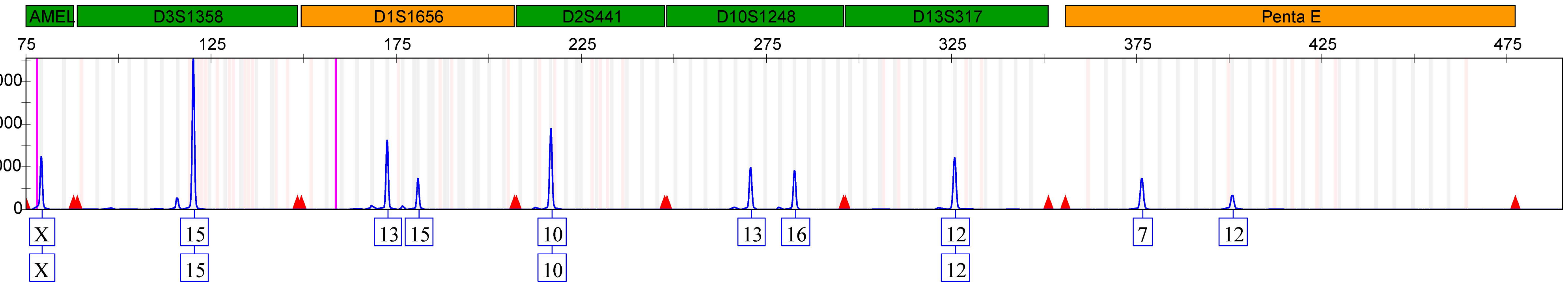

D-1060848_Cell Line_C-26696_F03_18.hid	D-1060848_Cell Line_C-26696						

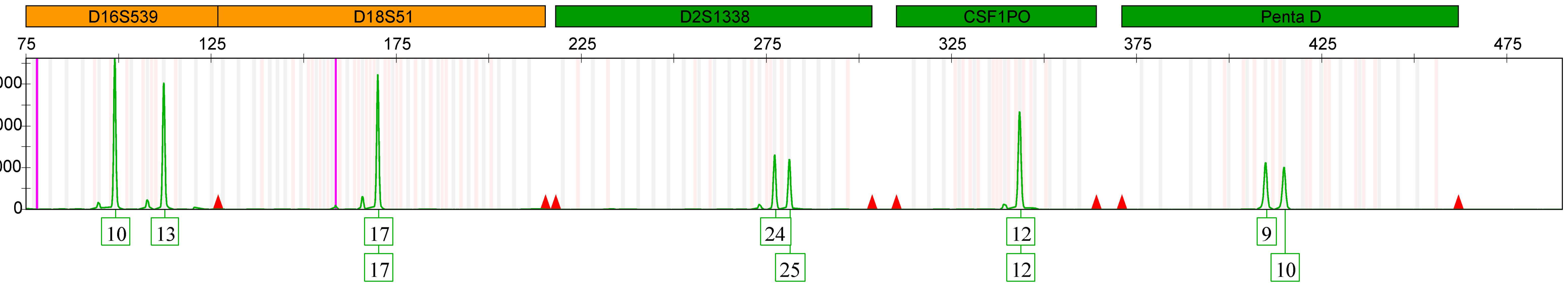

D-1060848_Cell Line_C-26696_F03_18.hid	D-1060848_Cell Line_C-26696						

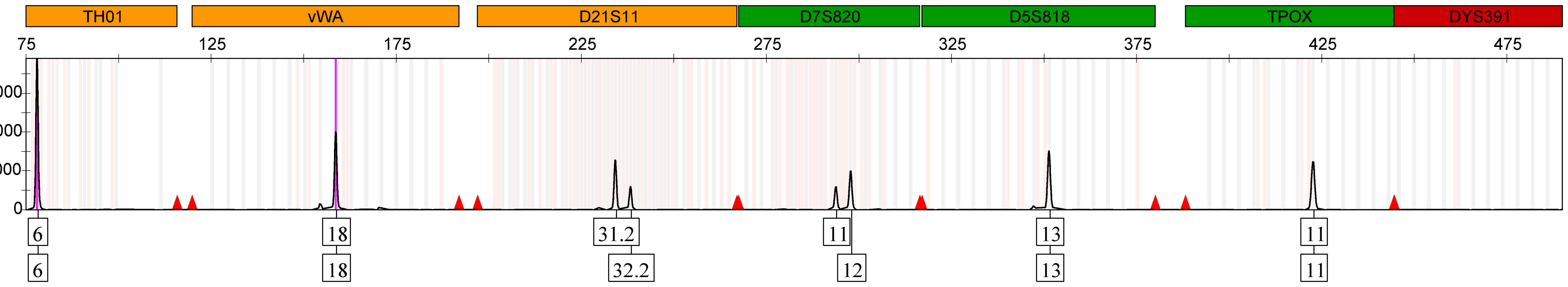

D-1060848_Cell Line_C-26696_F03_18.hid	D-1060848_Cell Line_C-26696						

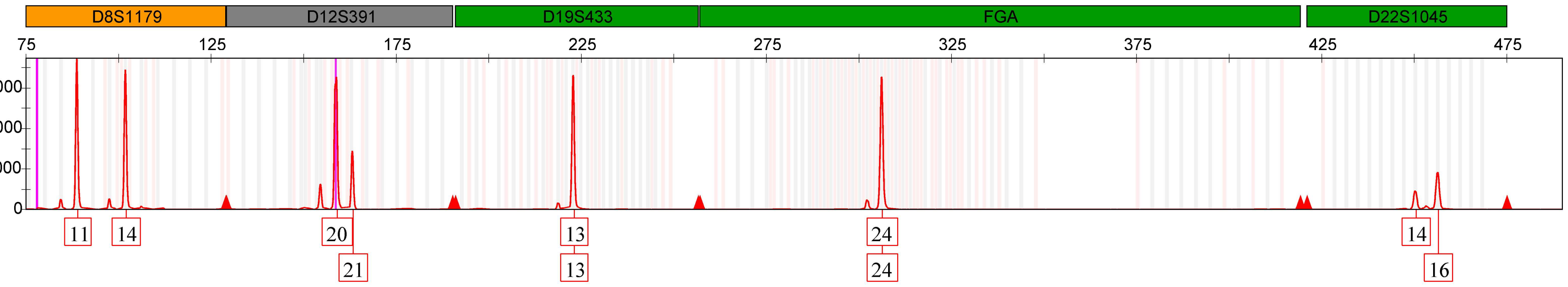

D-1060849_Cell Line_C-26696_G03_21.hid	D-1060849_Cell Line_C-26696						

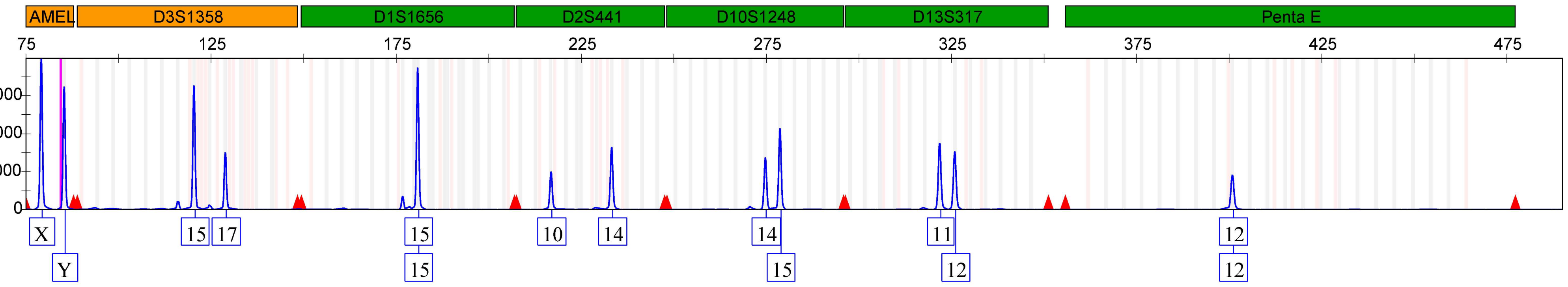

Sample File	Sample Name	SOS	SQ	SSPK	MIX	OMR	CGQ
D-1060849_Cell Line_C-26696_G03_21.hid	D-1060849_Cell Line_C-26696						

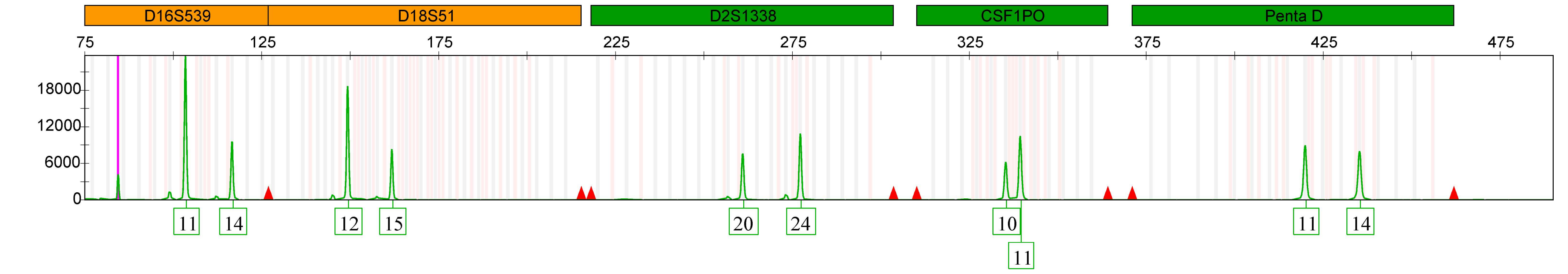

D-1060849_Cell Line_C-26696_G03_21.hid	D-1060849_Cell Line_C-26696						

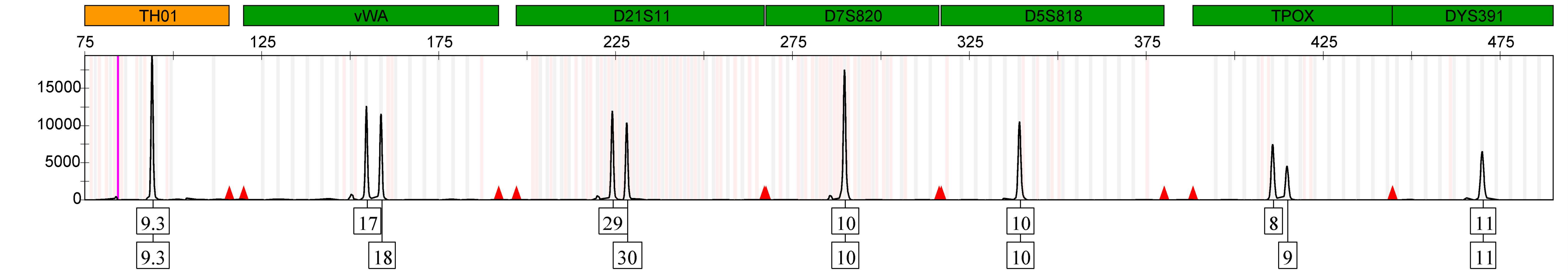

D-1060849_Cell Line_C-26696_G03_21.hid	D-1060849_Cell Line_C-26696						

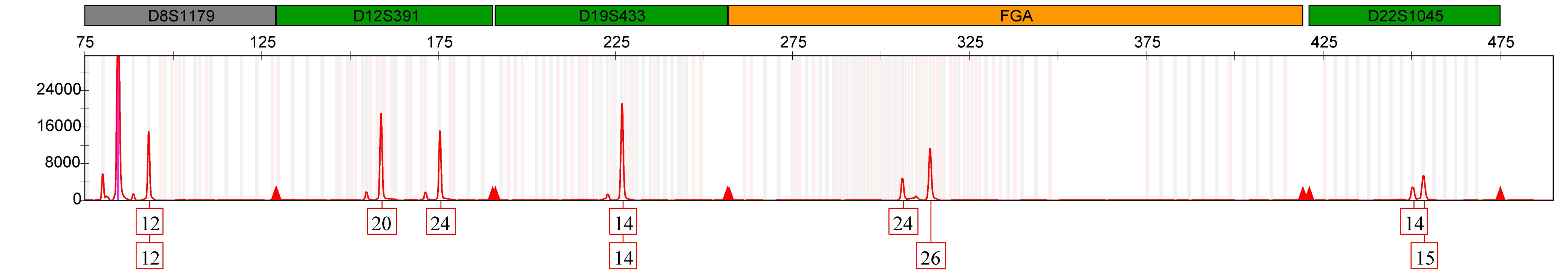

	Sample Name	Marker	Allele 1	Allele 2	Allele 3	Allele 4	Size 1	Size 2	Size 3	Size 4	Height 1	Height 2	Height 3	Height 4
1	Alleic_Ladder_2	AMEL	X	Y			79.09	85.31			4867	4568		
2	Alleic_Ladder_2	D3S1358	9	10	11	12	94.32	98.58	102.99	107.35	4507	4295	4216	4163
3	Alleic_Ladder_2	D1S1656	9	10	11	12	156.18	160.29	164.48	168.57	3700	3725	3768	3657
4	Alleic_Ladder_2	D2S441	8	9	10	11	208.57	212.68	216.81	220.84	3744	3628	3664	3761
5	Alleic_Ladder_2	D10S1248	8	9	10	11	250.42	254.49	258.57	262.54	3173	4109	3073	3077
6	Alleic_Ladder_2	D13S317	5	6	7	8	297.6	301.58	305.58	309.7	3493	3400	3503	3396
7	Alleic_Ladder_2	Penta E	5	6	7	8	366.78	371.6	376.34	381.27	3342	3286	3369	3367
8	Alleic_Ladder_2	D16S539	4	5	6	7	72.73	77.17	81.58	85.96	5616	5598	5403	5377
9	Alleic_Ladder_2	D18S51	7	8	9	10	128.17	132.4	136.63	140.88	4511	3666	4002	3853
10	Alleic_Ladder_2	D2S1338	10	12	14	15	220.04	228.16	236.25	240.37	3710	4253	4191	4286
11	Alleic_Ladder_2	CSF1PO	5	6	7	8	314.81	318.87	322.95	326.96	4393	4456	4344	4662
12	Alleic_Ladder_2	Penta D	2.2	3.2	5	6	376.45	381.49	389.71	394.79	4409	4265	4211	4211
13	Alleic_Ladder_2	TH01	3	4	5	6	64.7	69.27	73.66	77.99	5979	5969	5914	5984
14	Alleic_Ladder_2	vWA	10	11	12	13	125.28	129.61	133.74	137.88	5181	5123	5098	5037
15	Alleic_Ladder_2	D21S11	24	24.2	25	25.2	203.78	205.77	207.77	209.87	5078	4721	4861	4923
16	Alleic_Ladder_2	D7S820	5	6	7	8	269.55	273.64	277.69	281.72	4989	4915	4857	5019
17	Alleic_Ladder_2	D5S818	6	7	8	9	322.74	326.85	330.89	335.05	5061	5054	4931	4942
18	Alleic_Ladder_2	TPOX	4	5	6	7	394.67	398.64	402.67	406.68	4980	4928	4873	4869
19	Alleic_Ladder_2	DYS391	5	6	7	8	445.9	450.0	453.97	458.07	5159	5286	5346	5291
20	Alleic_Ladder_2	D8S1179	7	8	9	10	71.33	75.79	80.09	84.47	8759	8269	7853	7366
21	Alleic_Ladder_2	D12S391	14	15	16	17	133.74	137.98	142.14	146.33	6608	6370	6058	5842
22	Alleic_Ladder_2	D19S433	5.2	6.2	8	9	192.78	196.73	202.78	206.77	5932	6181	6092	5793
23	Alleic_Ladder_2	FGA	14	15	16	17	267.25	271.23	275.1	279.03	6095	5966	5987	5742
24	Alleic_Ladder_2	D22S1045	7	8	9	10	428.44	431.65	434.75	437.86	5699	5802	5741	5719
25	D-1060848_Cell Line_C-26696	AMEL	X	X			79.07	79.07			9964	9964		
26	D-1060848_Cell Line_C-26696	D3S1358	15	15			120.2	120.2			28375	28375		
27	D-1060848_Cell Line_C-26696	D1S1656	13	15			172.49	180.9			13060	5879		
28	D-1060848_Cell Line_C-26696	D2S441	10	10			216.8	216.8			15219	15219		
29	D-1060848_Cell Line_C-26696	D10S1248	13	16			270.68	282.64			7956	7247		
30	D-1060848_Cell Line_C-26696	D13S317	12	12			325.78	325.78			9729	9729		
31	D-1060848_Cell Line_C-26696	Penta E	7	12			376.39	400.8			6035	2860		
32	D-1060848_Cell Line_C-26696	D16S539	10	13			98.94	112.2			25354	21228		
33	D-1060848_Cell Line_C-26696	D18S51	17	17			169.99	169.99			22670	22670		
34	D-1060848_Cell Line_C-26696	D2S1338	24	25			277.23	281.16			9165	8420		
35	D-1060848_Cell Line_C-26696	CSF1PO	12	12			343.43	343.43			16391	16391		
36	D-1060848_Cell Line_C-26696	Penta D	9	10			409.87	414.84			7842	7076		
37	D-1060848_Cell Line_C-26696	TH01	6	6			77.96	77.96			31243	31243		
38	D-1060848_Cell Line_C-26696	vWA	18	18			158.6	158.6			16094	16094		
39	D-1060848_Cell Line_C-26696	D21S11	31.2	32.2			234.22	238.22			10236	4739		
40	D-1060848_Cell Line_C-26696	D7S820	11	12			293.71	297.76			4818	8007		
41	D-1060848_Cell Line_C-26696	D5S818	13	13			351.34	351.34			12161	12161		
42	D-1060848_Cell Line_C-26696	TPOX	11	11			422.65	422.65			10286	10286		
43	D-1060848_Cell Line_C-26696	DYS391												
44	D-1060848_Cell Line_C-26696	D8S1179	11	14			88.7	101.78			18656	17264		
45	D-1060848_Cell Line_C-26696	D12S391	20	21			158.8	163.09			16356	7260		
46	D-1060848_Cell Line_C-26696	D19S433	13	13			222.81	222.81			16566	16566		
47	D-1060848_Cell Line_C-26696	FGA	24	24			306.05	306.05			16339	16339		
48	D-1060848_Cell Line_C-26696	D22S1045	14	16			450.24	456.27			2453	4684		
49	D-1060849_Cell Line_C-26696	AMEL	X	Y			79.07	85.26			19923	16244		
50	D-1060849_Cell Line_C-26696	D3S1358	15	17			120.39	128.78			16313	7490		
51	D-1060849_Cell Line_C-26696	D1S1656	15	15			180.79	180.79			18710	18710		
52	D-1060849_Cell Line_C-26696	D2S441	10	14			216.77	233.21			4974	8198		
53	D-1060849_Cell Line_C-26696	D10S1248	14	15			274.68	278.66			6825	10734		
54	D-1060849_Cell Line_C-26696	D13S317	11	12			321.82	325.89			8684	7637		
55	D-1060849_Cell Line_C-26696	Penta E	12	12			400.91	400.91			4580	4580		
56	D-1060849_Cell Line_C-26696	D16S539	11	14			103.43	116.63			23668	9540		
57	D-1060849_Cell Line_C-26696	D18S51	12	15			149.25	161.77			18702	8292		
58	D-1060849_Cell Line_C-26696	D2S1338	20	24			260.95	277.2			7592	10882		
59	D-1060849_Cell Line_C-26696	CSF1PO	10	11			335.24	339.36			6217	10517		
60	D-1060849_Cell Line_C-26696	Penta D	11	14			419.9	435.25			8910	7960		
61	D-1060849_Cell Line_C-26696	TH01	9.3	9.3			93.98	93.98			19420	19420		
62	D-1060849_Cell Line_C-26696	vWA	17	18			154.57	158.72			12668	11590		
63	D-1060849_Cell Line_C-26696	D21S11	29	30			224.07	228.11			12020	10400		
64	D-1060849_Cell Line_C-26696	D7S820	10	10			289.68	289.68			17526	17526		
65	D-1060849_Cell Line_C-26696	D5S818	10	10			339.14	339.14			10559	10559		
66	D-1060849_Cell Line_C-26696	TPOX	8	9			410.7	414.71			7483	4552		
67	D-1060849_Cell Line_C-26696	DYS391	11	11			469.94	469.94			6526	6526		
68	D-1060849_Cell Line_C-26696	D8S1179	12	12			93.03	93.03			15083	15083		
69	D-1060849_Cell Line_C-26696	D12S391	20	24			158.72	175.36			19013	15207		
70	D-1060849_Cell Line_C-26696	D19S433	14	14			226.86	226.86			21226	21226		

Symbol key (added by the transcriber, not printed on the page): 🟩 = green square, ⚠ = yellow triangle, ⬜ = grey square, △ = grey triangle, 🛑 = red octagon, ⯃ = grey octagon.

	OS	BIN	PHR	LPH	MPH	SPK	AN	BD	CC	GQ
1	🟩	NA	NA	🟩	🟩	🟩	NA	🟩	NA	🟩
2	🟩	NA	NA	🟩	🟩	🟩	NA	🟩	NA	🟩
3	🟩	NA	NA	🟩	🟩	🟩	NA	🟩	NA	🟩
4	🟩	NA	NA	🟩	🟩	🟩	NA	🟩	NA	🟩
5	🟩	NA	NA	🟩	🟩	🟩	NA	🟩	NA	🟩
6	🟩	NA	NA	🟩	🟩	🟩	NA	🟩	NA	🟩
7	🟩	NA	NA	🟩	🟩	🟩	NA	🟩	NA	🟩
8	🟩	NA	NA	🟩	🟩	🟩	NA	🟩	NA	🟩
9	🟩	NA	NA	🟩	🟩	🟩	NA	🟩	NA	🟩
10	🟩	NA	NA	🟩	🟩	🟩	NA	🟩	NA	🟩
11	🟩	NA	NA	🟩	🟩	🟩	NA	🟩	NA	🟩
12	🟩	NA	NA	🟩	🟩	🟩	NA	🟩	NA	🟩
13	🟩	NA	NA	🟩	🟩	🟩	NA	🟩	NA	🟩
14	🟩	NA	NA	🟩	🟩	🟩	NA	🟩	NA	🟩
15	🟩	NA	NA	🟩	🟩	🟩	NA	🟩	NA	🟩
16	🟩	NA	NA	🟩	🟩	🟩	NA	🟩	NA	🟩
17	🟩	NA	NA	🟩	🟩	🟩	NA	🟩	NA	🟩
18	🟩	NA	NA	🟩	🟩	🟩	NA	🟩	NA	🟩
19	🟩	NA	NA	🟩	🟩	🟩	NA	🟩	NA	🟩
20	🟩	NA	NA	🟩	🟩	🟩	NA	🟩	NA	🟩
21	🟩	NA	NA	🟩	🟩	🟩	NA	🟩	NA	🟩
22	🟩	NA	NA	🟩	🟩	🟩	NA	🟩	NA	🟩
23	🟩	NA	NA	🟩	🟩	🟩	NA	🟩	NA	🟩
24	🟩	NA	NA	🟩	🟩	🟩	NA	🟩	NA	🟩
25	🟩	🟩	NA	🟩	🟩	🟩	🟩	🟩	NA	🟩
26	🟩	🟩	NA	🟩	🟩	🟩	🟩	🟩	NA	🟩
27	⚠	🟩	⚠	🟩	🟩	🟩	🟩	🟩	NA	⚠
28	🟩	🟩	NA	🟩	🟩	🟩	🟩	🟩	NA	🟩
29	🟩	🟩	🟩	🟩	🟩	🟩	🟩	🟩	NA	🟩
30	🟩	🟩	NA	🟩	🟩	🟩	🟩	🟩	NA	🟩
31	🟩	🟩	⚠	🟩	🟩	🟩	🟩	🟩	NA	⚠
32	⚠	🟩	🟩	🟩	🟩	🟩	🟩	🟩	NA	⚠
33	⚠	🟩	NA	🟩	🟩	🟩	🟩	🟩	NA	⚠
34	🟩	🟩	🟩	🟩	🟩	🟩	🟩	🟩	NA	🟩
35	🟩	🟩	NA	🟩	🟩	🟩	🟩	🟩	NA	🟩
36	🟩	🟩	🟩	🟩	🟩	🟩	🟩	🟩	NA	🟩
37	⚠	🟩	NA	🟩	NA	🟩	🟩	🟩	NA	⚠
38	⚠	🟩	NA	🟩	NA	🟩	🟩	🟩	NA	⚠
39	🟩	🟩	⚠	🟩	🟩	🟩	🟩	🟩	NA	⚠
40	🟩	🟩	🟩	🟩	🟩	🟩	🟩	🟩	NA	🟩
41	🟩	🟩	NA	🟩	🟩	🟩	🟩	🟩	NA	🟩
42	🟩	🟩	NA	🟩	🟩	🟩	🟩	🟩	NA	🟩
43	🟩	NA	NA	NA	NA	🟩	⚠	NA	NA	🛑
44	⚠	🟩	🟩	🟩	🟩	🟩	🟩	🟩	NA	⚠
45	△	⬜	△	⬜	⬜	⬜	△	⬜	NA	⯃
46	🟩	🟩	NA	🟩	🟩	🟩	🟩	🟩	NA	🟩
47	🟩	🟩	NA	🟩	🟩	🟩	🟩	🟩	NA	🟩
48	🟩	🟩	🟩	🟩	🟩	🟩	🟩	🟩	NA	🟩
49	⚠	🟩	🟩	🟩	🟩	🟩	🟩	🟩	NA	⚠
50	🟩	🟩	⚠	🟩	🟩	🟩	🟩	🟩	NA	⚠
51	🟩	🟩	NA	🟩	🟩	🟩	🟩	🟩	NA	🟩
52	🟩	🟩	🟩	🟩	🟩	🟩	🟩	🟩	NA	🟩
53	🟩	🟩	🟩	🟩	🟩	🟩	🟩	🟩	NA	🟩
54	🟩	🟩	🟩	🟩	🟩	🟩	🟩	🟩	NA	🟩
55	🟩	🟩	NA	🟩	🟩	🟩	🟩	🟩	NA	🟩
56	⚠	🟩	⚠	🟩	🟩	🟩	🟩	🟩	NA	⚠
57	🟩	🟩	⚠	🟩	🟩	🟩	🟩	🟩	NA	⚠
58	🟩	🟩	🟩	🟩	🟩	🟩	🟩	🟩	NA	🟩
59	🟩	🟩	🟩	🟩	🟩	🟩	🟩	🟩	NA	🟩
60	🟩	🟩	🟩	🟩	🟩	🟩	🟩	🟩	NA	🟩
61	⚠	🟩	NA	🟩	🟩	🟩	🟩	🟩	NA	⚠
62	🟩	🟩	🟩	🟩	🟩	🟩	🟩	🟩	NA	🟩
63	🟩	🟩	🟩	🟩	🟩	🟩	🟩	🟩	NA	🟩
64	🟩	🟩	NA	🟩	🟩	🟩	🟩	🟩	NA	🟩
65	🟩	🟩	NA	🟩	🟩	🟩	🟩	🟩	NA	🟩
66	🟩	🟩	🟩	🟩	🟩	🟩	🟩	🟩	NA	🟩
67	🟩	🟩	NA	🟩	🟩	🟩	🟩	🟩	NA	🟩
68	⬜	⬜	△	⬜	⬜	⬜	⬜	⬜	NA	△
69	🟩	🟩	🟩	🟩	🟩	🟩	🟩	🟩	NA	🟩
70	🟩	🟩	NA	🟩	🟩	🟩	🟩	🟩	NA	🟩

	Sample Name	Marker	Allele 1	Allele 2	Allele 3	Allele 4	Size 1	Size 2	Size 3	Size 4	Height 1	Height 2	Height 3	Height 4
71	D-1060849_Cell Line_C-26696	FGA	24	26			306.1	313.87			4914	11394		
72	D-1060849_Cell Line_C-26696	D22S1045	14	15			450.36	453.32			3185	5508		

	OS	BIN	PHR	LPH	MPH	SPK	AN	BD	CC	GQ
71	■	■	▲	■	■	■	■	■	NA	▲
72	■	■	■	■	■	■	■	■	NA	■

Sample File	Sample Name	SOS	SQ	SSPK	MIX	OMR	CGQ
Allele Ladder 3 H06 24.hid	Allele Ladder 3				NA	NA	

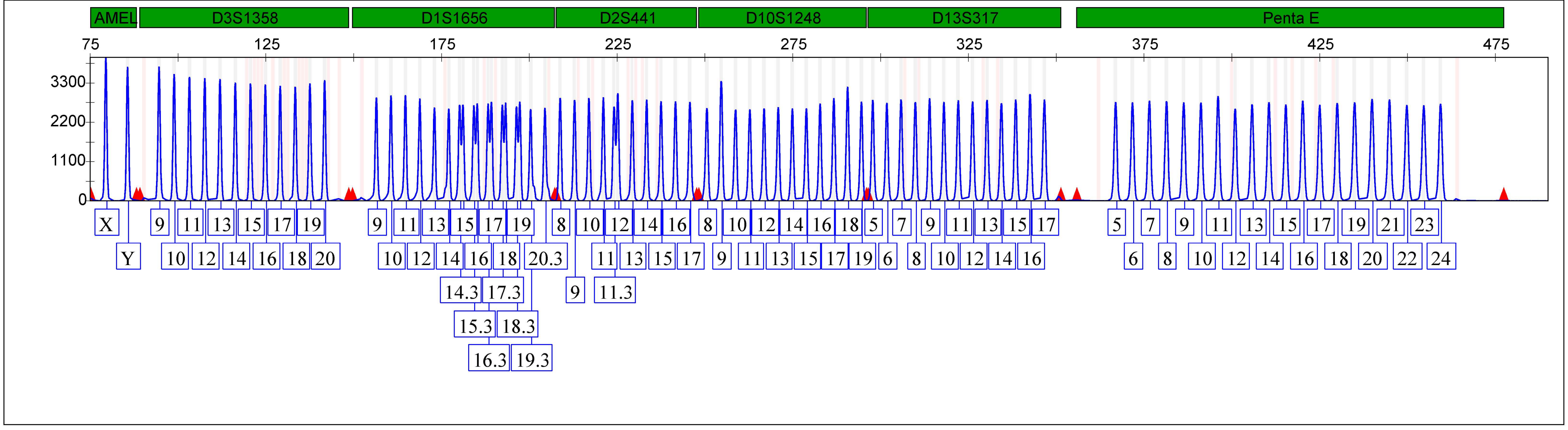

Allele Ladder 3 H06 24.hid	Allele Ladder 3				NA	NA	

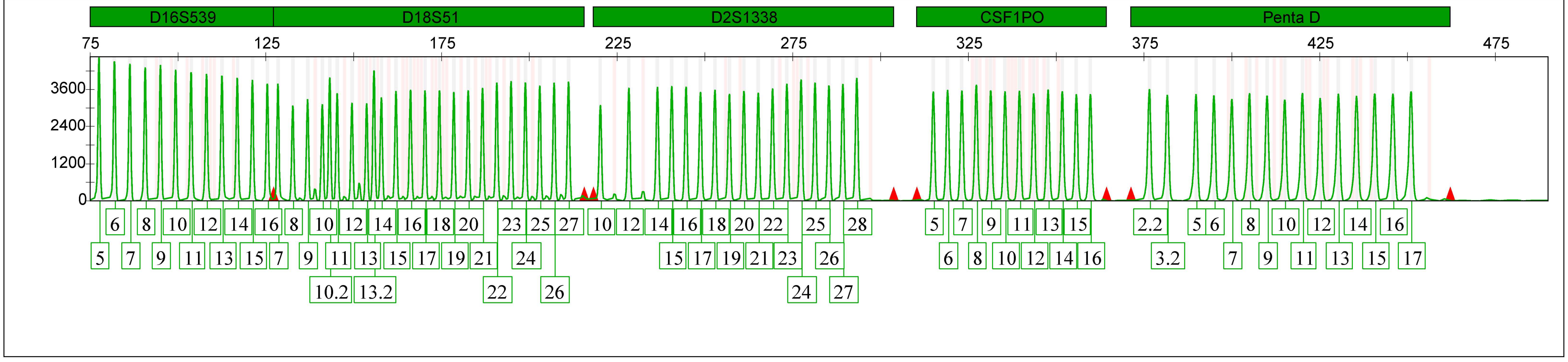

Allele Ladder 3 H06 24.hid	Allele Ladder 3				NA	NA	

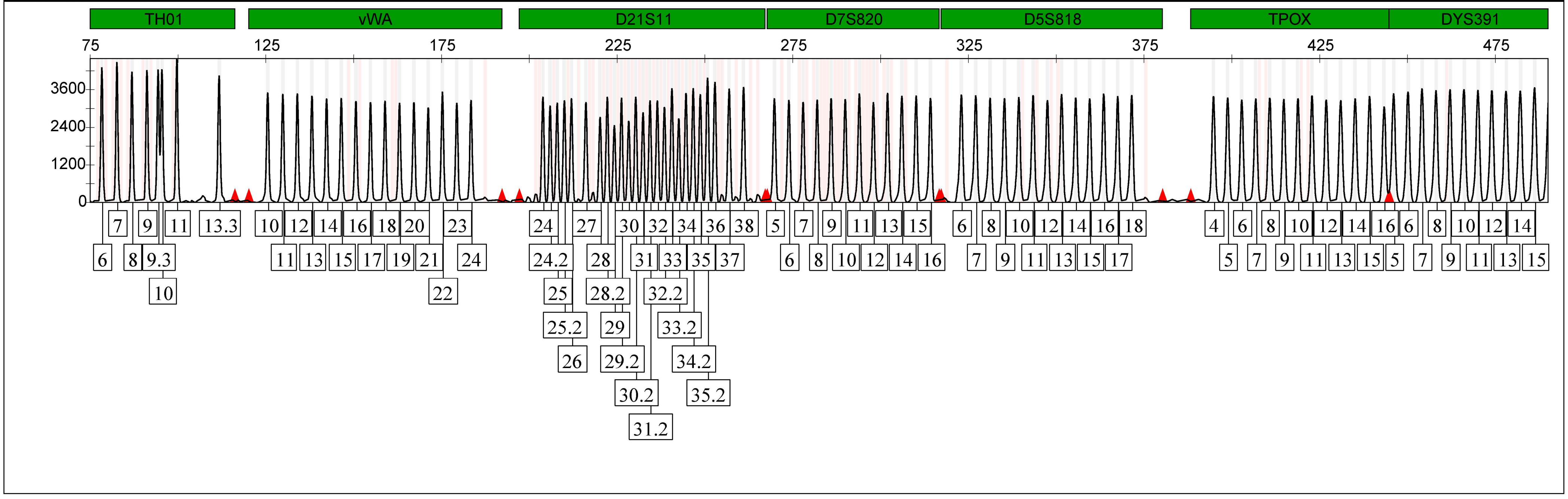

Allele Ladder 3 H06 24.hid	Allele Ladder 3				NA	NA	

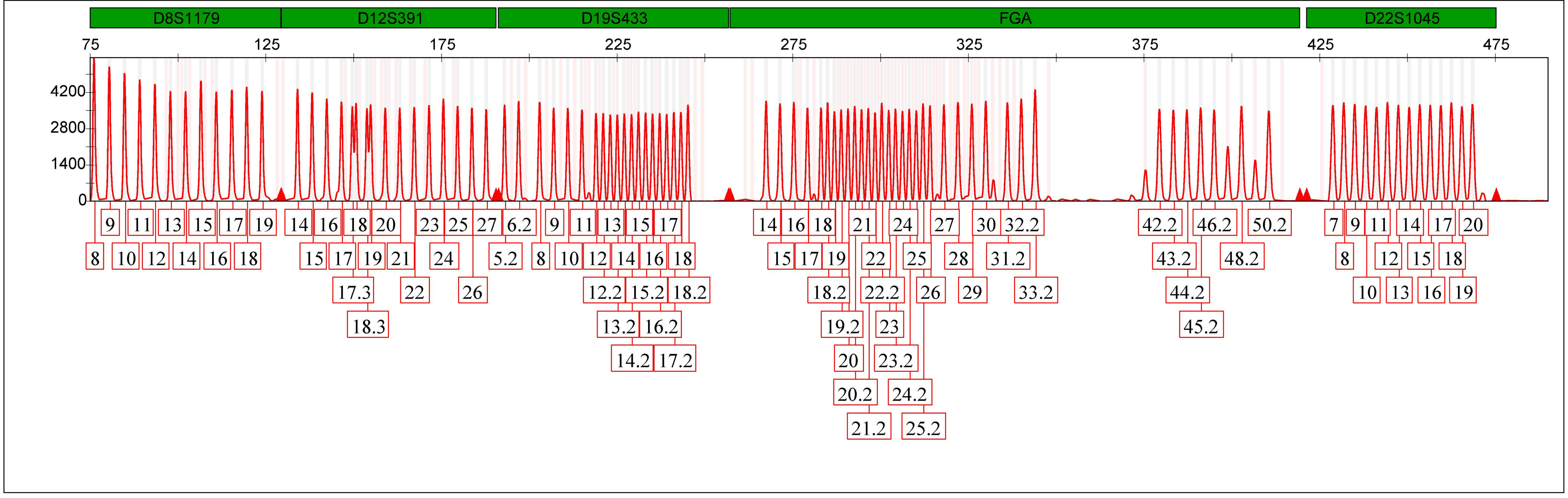

Applied Biosystems
GeneMapper® ID-X 1.5

Project: LW10100309-22

Sample File	Sample Name	SOS	SQ	SSPK	MIX	OMR	CGQ
D-1061347_C-26872_Cell Line_C06_09.hid	D-1061347_C-26872_Cell Line						

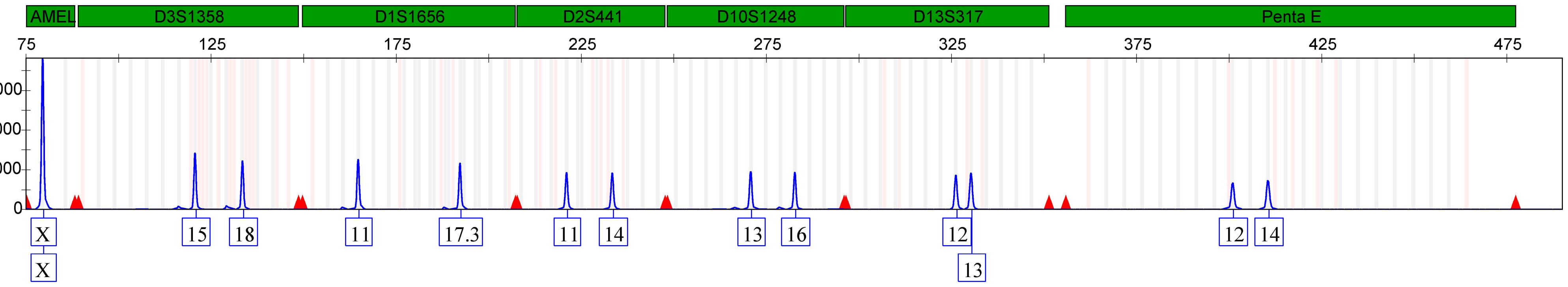

| D-1061347_C-26872_Cell Line_C06_09.hid | D-1061347_C-26872_Cell Line | | | | | | |

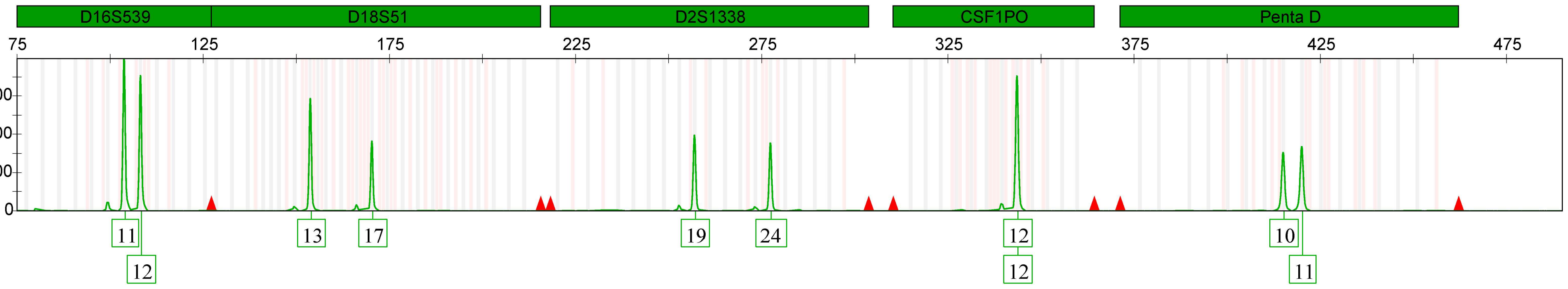

| D-1061347_C-26872_Cell Line_C06_09.hid | D-1061347_C-26872_Cell Line | | | | | | |

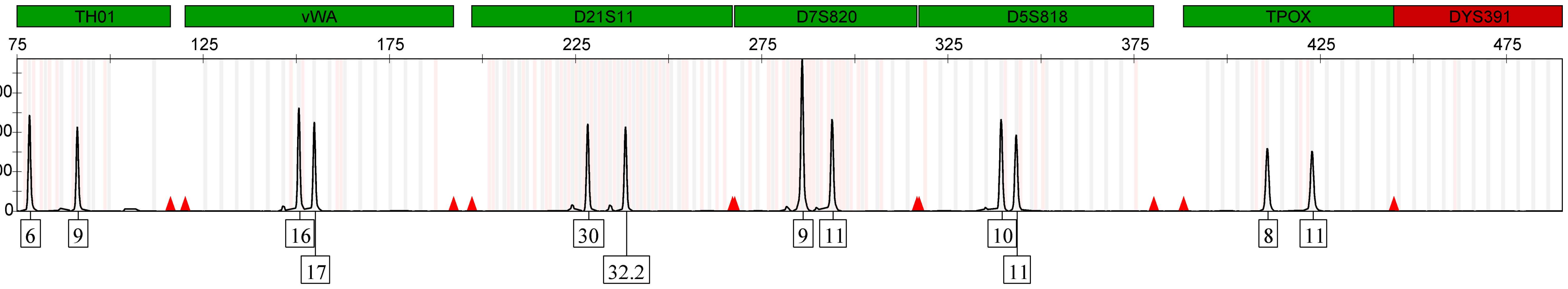

| D-1061347_C-26872_Cell Line_C06_09.hid | D-1061347_C-26872_Cell Line | | | | | | |

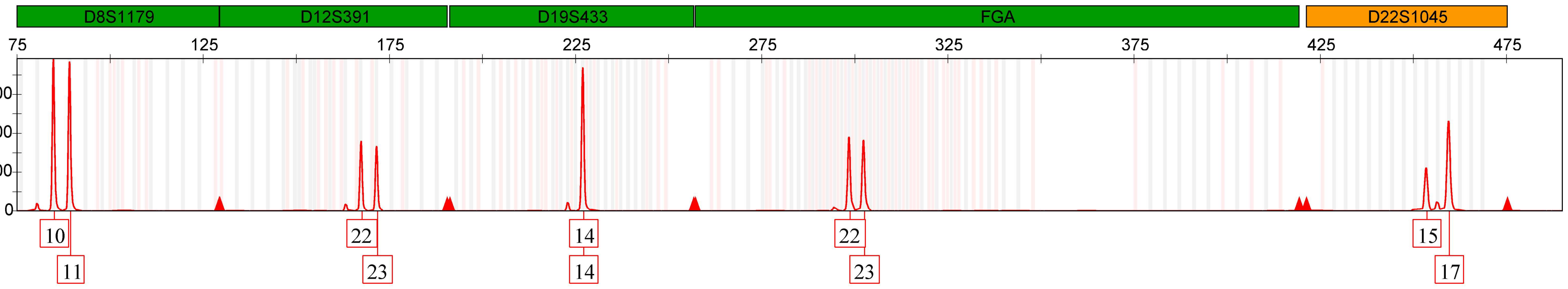

	Sample Name	Marker	Allele 1	Allele 2	Allele 3	Allele 4	Size 1	Size 2	Size 3	Size 4	Height 1	Height 2	Height 3	Height 4
1	D-1061347_C-26872_Cell Line	AMEL	X	X			79.45	79.45			15245	15245		
2	D-1061347_C-26872_Cell Line	D3S1358	15	18			120.67	133.46			5671	4913		
3	D-1061347_C-26872_Cell Line	D1S1656	11	17.3			164.65	192.18			5025	4676		
4	D-1061347_C-26872_Cell Line	D2S441	11	14			220.96	233.37			3752	3653		
5	D-1061347_C-26872_Cell Line	D10S1248	13	16			270.7	282.65			3813	3765		
6	D-1061347_C-26872_Cell Line	D13S317	12	13			326.19	330.22			3455	3699		
7	D-1061347_C-26872_Cell Line	Penta E	12	14			400.89	410.5			2721	2998		
8	D-1061347_C-26872_Cell Line	D16S539	11	12			103.77	108.12			8349	7457		
9	D-1061347_C-26872_Cell Line	D18S51	13	17			153.78	170.27			6178	3839		
10	D-1061347_C-26872_Cell Line	D2S1338	19	24			256.89	277.27			4153	3722		
11	D-1061347_C-26872_Cell Line	CSF1PO	12	12			343.53	343.53			7431	7431		
12	D-1061347_C-26872_Cell Line	Penta D	10	11			415.0	419.99			3214	3540		
13	D-1061347_C-26872_Cell Line	TH01	6	9			78.35	91.16			5377	4713		
14	D-1061347_C-26872_Cell Line	vWA	16	17			150.67	154.85			5784	4962		
15	D-1061347_C-26872_Cell Line	D21S11	30	32.2			228.26	238.41			4863	4707		
16	D-1061347_C-26872_Cell Line	D7S820	9	11			285.86	293.85			8516	5111		
17	D-1061347_C-26872_Cell Line	D5S818	10	11			339.27	343.31			5122	4258		
18	D-1061347_C-26872_Cell Line	TPOX	8	11			410.72	422.83			3504	3334		
19	D-1061347_C-26872_Cell Line	DYS391												
20	D-1061347_C-26872_Cell Line	D8S1179	10	11			84.73	89.11			9390	9212		
21	D-1061347_C-26872_Cell Line	D12S391	22	23			167.36	171.54			4323	4015		
22	D-1061347_C-26872_Cell Line	D19S433	14	14			226.93	226.93			8868	8868		
23	D-1061347_C-26872_Cell Line	FGA	22	23			298.44	302.32			4580	4390		
24	D-1061347_C-26872_Cell Line	D22S1045	15	17			453.39	459.38			2669	5539		
25	Allele Ladder 3	AMEL	X	Y			79.45	85.64			4013	3751		
26	Allele Ladder 3	D3S1358	9	10	11	12	94.57	98.87	103.27	107.6	3758	3553	3465	3438
27	Allele Ladder 3	D1S1656	9	10	11	12	156.49	160.58	164.75	168.83	2881	2944	2951	2862
28	Allele Ladder 3	D2S441	8	9	10	11	208.73	212.83	216.94	221.06	2874	2812	2868	2901
29	Allele Ladder 3	D10S1248	8	9	10	11	250.52	254.58	258.74	262.8	2588	3341	2552	2554
30	Allele Ladder 3	D13S317	5	6	7	8	297.73	301.79	305.79	309.92	2818	2737	2833	2760
31	Allele Ladder 3	Penta E	5	6	7	8	366.95	371.73	376.56	381.34	2757	2752	2797	2770
32	Allele Ladder 3	D16S539	4	5	6	7	72.99	77.52	81.85	86.29	4690	4663	4509	4440
33	Allele Ladder 3	D18S51	7	8	9	10	128.42	132.63	136.84	141.06	3782	3075	3283	3127
34	Allele Ladder 3	D2S1338	10	12	14	15	220.25	228.34	236.47	240.55	3090	3655	3683	3707
35	Allele Ladder 3	CSF1PO	5	6	7	8	315.02	319.09	323.17	327.27	3526	3582	3558	3744
36	Allele Ladder 3	Penta D	2.2	3.2	5	6	376.56	381.57	389.74	394.92	3606	3420	3439	3407
37	Allele Ladder 3	TH01	3	4	5	6	65.0	69.53	73.92	78.26	4382	4364	4226	4315
38	Allele Ladder 3	vWA	10	11	12	13	125.55	129.85	133.97	138.08	3508	3456	3474	3396
39	Allele Ladder 3	D21S11	24	24.2	25	25.2	203.86	205.95	207.94	209.93	3373	3093	3184	3250
40	Allele Ladder 3	D7S820	5	6	7	8	269.78	273.85	277.87	281.97	3321	3271	3190	3276
41	Allele Ladder 3	D5S818	6	7	8	9	322.95	327.06	331.18	335.3	3434	3412	3335	3320
42	Allele Ladder 3	TPOX	4	5	6	7	394.8	398.76	402.78	406.8	3380	3340	3276	3313
43	Allele Ladder 3	DYS391	5	6	7	8	446.06	450.12	454.06	458.12	3473	3531	3622	3574
44	Allele Ladder 3	D8S1179	7	8	9	10	71.69	76.05	80.46	84.8	5962	5539	5192	4934
45	Allele Ladder 3	D12S391	14	15	16	17	133.97	138.18	142.32	146.49	4332	4207	3951	3829
46	Allele Ladder 3	D19S433	5.2	6.2	8	9	192.94	196.96	202.97	206.94	3722	3858	3811	3599
47	Allele Ladder 3	FGA	14	15	16	17	267.39	271.35	275.31	279.2	3866	3772	3820	3597
48	Allele Ladder 3	D22S1045	7	8	9	10	428.76	431.83	434.9	438.1	3707	3806	3744	3690

	OS	BIN	PHR	LPH	MPH	SPK	AN	BD	CC	GQ
1	■	■	NA	■	■	■	■	■	NA	■
2	■	■	■	■	■	■	■	■	NA	■
3	■	■	■	■	■	■	■	■	NA	■
4	■	■	■	■	■	■	■	■	NA	■
5	■	■	■	■	■	■	■	■	NA	■
6	■	■	■	■	■	■	■	■	NA	■
7	■	■	■	■	■	■	■	■	NA	■
8	■	■	■	■	■	■	■	■	NA	■
9	■	■	■	■	■	■	■	■	NA	■
10	■	■	■	■	■	■	■	■	NA	■
11	■	■	NA	■	■	■	■	■	NA	■
12	■	■	■	■	■	■	■	■	NA	■
13	■	■	■	■	■	■	■	■	NA	■
14	■	■	■	■	■	■	■	■	NA	■
15	■	■	■	■	■	■	■	■	NA	■
16	■	■	■	■	■	■	■	■	NA	■
17	■	■	■	■	■	■	■	■	NA	■
18	■	■	■	■	■	■	■	■	NA	■
19	■	NA	NA	NA	NA	■	▲	NA	NA	⬣
20	■	■	■	■	■	■	■	■	NA	■
21	■	■	■	■	■	■	■	■	NA	■
22	■	■	NA	■	■	■	■	■	NA	■
23	■	■	■	■	■	■	■	■	NA	■
24	■	■	▲	■	■	■	■	■	NA	▲
25	■	NA	NA	■	■	■	NA	■	NA	■
26	■	NA	NA	■	■	■	NA	■	NA	■
27	■	NA	NA	■	■	■	NA	■	NA	■
28	■	NA	NA	■	■	■	NA	■	NA	■
29	■	NA	NA	■	■	■	NA	■	NA	■
30	■	NA	NA	■	■	■	NA	■	NA	■
31	■	NA	NA	■	■	■	NA	■	NA	■
32	■	NA	NA	■	■	■	NA	■	NA	■
33	■	NA	NA	■	■	■	NA	■	NA	■
34	■	NA	NA	■	■	■	NA	■	NA	■
35	■	NA	NA	■	■	■	NA	■	NA	■
36	■	NA	NA	■	■	■	NA	■	NA	■
37	■	NA	NA	■	■	■	NA	■	NA	■
38	■	NA	NA	■	■	■	NA	■	NA	■
39	■	NA	NA	■	■	■	NA	■	NA	■
40	■	NA	NA	■	■	■	NA	■	NA	■
41	■	NA	NA	■	■	■	NA	■	NA	■
42	■	NA	NA	■	■	■	NA	■	NA	■
43	■	NA	NA	■	■	■	NA	■	NA	■
44	■	NA	NA	■	■	■	NA	■	NA	■
45	■	NA	NA	■	■	■	NA	■	NA	■
46	■	NA	NA	■	■	■	NA	■	NA	■
47	■	NA	NA	■	■	■	NA	■	NA	■
48	■	NA	NA	■	■	■	NA	■	NA	■
